# Regulation and Function of Metal Uptake Transporter NtNRAMP3 in Tobacco

**DOI:** 10.3389/fpls.2022.867967

**Published:** 2022-05-31

**Authors:** Katarzyna Kozak, Anna Papierniak-Wygladala, Małgorzata Palusińska, Anna Barabasz, Danuta Maria Antosiewicz

**Affiliations:** Faculty of Biology, Institute of Experimental Plant Biology and Biotechnology, University of Warsaw, Warsaw, Poland

**Keywords:** NtNRAMP3, tobacco, metal uptake, iron, zinc, manganese, cobalt, nickel

## Abstract

Natural resistance-associated macrophage protein (*NRAMP*) genes encode proteins with low substrate specificity, important for maintaining metal cross homeostasis in the cell. The role of these proteins in tobacco, an important crop plant with wide application in the tobacco industry as well as in phytoremediation of metal-contaminated soils, remains unknown. Here, we identified NtNRAMP3, the closest homologue to NRAMP3 proteins from other plant species, and functionally characterized it. A NtNRAMP3-GFP fusion protein was localized to the plasma membrane in tobacco epidermal cells. Expression of *NtNRAMP3* in yeast was able to rescue the growth of Fe and Mn uptake defective Δ*fet3fet4* and Δ*smf1* mutant yeast strains, respectively. Furthermore, *NtNRAMP3* expression in wild-type *Saccharomyces cerevisiae* DY1457 yeast strain increased sensitivity to elevated concentrations of iron (Fe), manganese (Mn), copper (Cu), cobalt (Co), nickel (Ni), and cadmium (Cd). Taken together, these results point to a possible role in the uptake of metals. NtNRAMP3 was expressed in the leaves and to a lesser extent in the roots of tobacco plants. Its expression occurred mainly under control conditions and decreased very sharply in deficiency and excess of the tested metals. GUS-based analysis of the site-specific activity of the *NtNRAMP3* promoter showed that it was primarily expressed in the xylem of leaf blades. Overall, our data indicate that the main function of NtNRAMP3 is to maintain cross homeostasis of Fe, Mn, Co, Cu, and Ni (also Cd) in leaves under control conditions by controlling xylem unloading.

## Introduction

Excess metals taken up from the soil are accumulated by most plants in their leaves, however, the efficiency of accumulation in these organs differs among them. Those capable of storing very large amounts are frequently used in the phytoremediation of metal-contaminated soil. Tobacco is one such species. It is a high biomass plant effective in translocating metal from the root to the shoot, especially zinc (Zn) and cadmium (Cd), which is an important determinant of accumulation in leaves (Wagner and Yeargan, 1986; Angelova et al., 2004; Doroszewska and Berbeć, 2004; Lugon-Moulin et al., 2004). Furthermore, many attempts have been made to increase its usefulness in phytoremediation through genetic modification (Wojas et al., 2009; Grispen et al., 2011; Das et al., 2016; Rehman et al., 2019). Nevertheless, little is known about the mechanisms of metal tolerance and the accumulation of high concentrations of metals in tobacco leaves. Knowledge of these processes is not only epistemic but also application-related for the use of tobacco in cleaning up contaminated soil.

One of the key factors in a plant's tolerance to Zn is the ability to store metal in leaves without detrimental effects. A commonly accepted symptom of Zn-sensitivity is the development of necrotic regions over the leaf blades, therefore, monitoring their appearance upon exposure to high Zn became a tool to assess sensitivity to excess Zn (Marschner, 1995). Our recent study demonstrated that in leaves of plants exposed to high Zn, necrotic regions develop from groups of mesophyll cells (“Zn accumulating cells”) that contain very high Zn concentrations. They develop necrosis when the Zn concentration exceeds a certain threshold, and programmed cell death (PCD) is involved in this process (Siemianowski et al., 2013; Weremczuk et al., 2017, 2020). The neighboring, non-accumulating cells, do not experience high internal Zn. Such functional diversification of the ability to accumulate Zn at the cellular level in the leaf mesophyll was suggested to be crucial in maintaining the function of the whole leaf at high Zn exposure. This is achieved by mechanisms staving off toxic levels of Zn incertain cells (non-accumulating ones), thereby allowing them to continue with their basic functions. Thus, contrary to the general view, our data indicate that the development of necrosis resulting from the differentiation of mesophyll cells in terms of Zn accumulation can be considered a defense mechanism rather than merely a manifestation of Zn toxicity (Siemianowski et al., 2013).

In our previous research aimed at elucidating the molecular basis of the differential ability of mesophyll cells to accumulate Zn, several candidate genes were identified, including *NtZIP1-like, NtZIP11*, and *NtNRAMP3-like* (Papierniak et al., 2018). Subsequently, a detailed study of two of them, *NtZIP1-like* and *NtZIP11* (Zrt-Irt-like Proteins), showed that they likely participate in the cell-specific accumulation of Zn in the palisade parenchyma of tobacco leaves (Kozak et al., 2019; Papierniak-Wygladala et al., 2020). The third identified gene, *NtNRAMP3-like*, was shown to be highly upregulated shortly after administration of an elevated Zn concentration (200 μM), similarly to *NtZIP1-like* and *NtZIP11* (Papierniak et al., 2018), was considered a candidate that may also play a role in the distinct capacity of mesophyll cells to accumulate Zn. It is known that the accumulation of metals involves a range of gene encoding proteins responsible for their uptake and removal outside of the cell, loading into the vacuole, or exporting to the cytoplasm.

The NRAMP family includes proton-coupled metal ion transporters that mediate the transport of a broad range of metal ions, such as manganese (Mn^2+^), Zn^2+^, copper (Cu^2+^), iron (Fe^2+^), Cd^2+^, nickel (Ni^2+^), and cobalt (Co^2+^), into the cytoplasm (Colangelo and Guerinot, 2006; Nevo and Nelson, 2006). The physiological role of NRAMPs in plants is not well-understood. Current research indicates diverse functions, depending on the gene, its regulation, the subcellular localization of the encoded protein, and its substrates (Legay et al., 2012; Qin et al., 2017; Tian et al., 2021). The NRAMPs were shown to participate in the uptake of essential metals, e.g., Fe, Mn, and Zn (Thomine et al., 2000; Cailliatte et al., 2010; Sasaki et al., 2012; Xiong et al., 2012; Yamaji et al., 2013; Tiwari et al., 2014; Zhang et al., 2020). They also affect subcellular redistribution localized in the tonoplast or the intracellular vesicles (Bereczky et al., 2003; Thomine et al., 2003; Languar et al., 2005; Li et al., 2019; Pottier et al., 2022). Several NRAMPs were also shown to be involved in the uptake of toxic Cd (Cailliatte et al., 2010; Takahashi et al., 2011) or its redistribution (Thomine et al., 2003; Languar et al., 2004).

Although the expression of most *NRAMP* genes was induced under mineral deficiency conditions, some showed an increase in transcript levels under stress generated by the presence of metal excess. For example, elevated expression at 100 μM Zn was detected in the shoots of *Thlaspi caerulescens* for *TcNRAMP3* and *TcNRAMP4* (Oomen et al., 2009), and for *TcNRAMP4* in the presence of 1,000 μM ferrozine or 600 μM Ni (Wei et al., 2009). Several soybean *NRAMPs* were also shown to be upregulated by toxic 200 μM Cu (*GmNRAMP1a*; *3a* and *5a*), 100 μM Cd (GmNRAMP1a, 1b, 3a, 5a), 1,000 μM Fe/EDTA (*GmNRAMP6a*), or 200 μM Mn (*GmNRAMP5a*) (Qin et al., 2017). Upregulation by unessential toxic Cd was also noted for *MhNRAMP1* (Zhang et al., 2020), *TcNRAMP3* (Wei et al., 2009), or *StNRAMP3* (Tian et al., 2021). Furthermore, the expression level of *NRAMP3* was also higher in Zn/Cd hyperaccumulator *A. halleri* than in *A. thaliana* (Weber et al., 2004). Therefore, participation of NRAMPs in regulating the response to high concentrations of metals seems certain, but it is not known what this role is.

Based on the results of preliminary studies on the *NtNRAMP3*-*like* sequence (increased expression in the presence of 200 Zn in leaves; Papierniak et al., 2018), and literature data, it was hypothesized that *NtNRAMP3-like*, in addition to *NtZIP1-like* and *NtZIP11*, may be another component of a network of processes regulating metal homeostasis in tobacco leaves exposed to excess Zn, including cell-specific accumulation of this metal in the leaf blades. In this work, we cloned *NtNRAMP3* and determined its metal transport activity and biological role in tobacco.

## Materials and Methods

### Plant Material, Growth Conditions, and Experimental Design

Wild-type (WT) tobacco *Nicotiana tabacum* var. Xanthi and generated tobacco transgenic plants expressing *pMDC163::promNtNRAMP3::GUS* were used in the study. Seeds of the WT tobacco were obtained from the stock of the Institute of Biochemistry and Biophysics PAS (Warsaw, Poland; in 2002) and then propagated in the greenhouse of the University of Warsaw.

Plants were cultivated in a growth chamber at 23/16°C day/night, 40–50% humidity, 16 h photoperiod, and quantum flux density [photosynthetically active radiation (PAR)] 250 μMol m^−2^ s^−1^, fluorescent Flora tubes (Siemianowski et al., 2011).

Seeds were surface sterilized in 8% sodium hypochlorite (w/v) for 2 min, washed with distilled water, then germinated and grown on vertically positioned Petri dishes containing quarter-strength Knop's medium, 2% sucrose (w/v), and 1% agar (w/v). After 3 weeks seedlings were transferred to hydroponic conditions (five plants per 2.5 L pot) and further grown according to experiment-specific schemes presented below. In all experiments, the quarter-strength Knop's medium was applied as a reference control medium (Barabasz et al., 2010). All experiments were conducted in three independent biological replicates. The nutrient solution was renewed every third day.

To determine developmental regulation of *NtNRAMP3*, 3-week-old WT seedlings were transferred from the Petri dishes into the hydroponic control medium for up to 5.5 weeks. Plant material was collected at three developmental stages: (1) 4-week-old seedlings (3 weeks on plates and 1 week on hydroponics): whole roots and all leaves were collected separately; (2) 6-week-old plants (3 weeks on plates and 3 weeks on hydroponics): whole roots and all leaves were collected separately; (3) 9-week-old plants (3 weeks on plates and 6 weeks on hydroponics); the following plant parts were collected: (a) apical part of the root (3–4 cm from the tip), (b) basal part of the root (3–4 cm from the base), (c) stem (3 cm of the middle part), (d) young leaves (two leaves of minimum 0.5 cm length counting from the top), and (e) old leaves (two leaves counting from the base). Lateral roots were not included in the analysis. The material was pooled from a total of 30 (stage 1), 15 (stage 2), or 10 (stage 3) plants, frozen in the liquid nitrogen and stored at −80°C.

For assessment of metal status-dependent expression of *NtNRAMP3*, 3-week-old WT plants were further hydroponically grown on a control medium for 2 weeks. Then, they were exposed to different regimes: (i) metal excess (200 μM Fe or 100 μM Mn or 20 μM Co or 20 μM Cu or 30 μM Ni or 50 μM Zn or 4 μM Cd); (ii) metal deficiency (Fe or Mn or Co or Cu or Zn; metal was not added to the medium); (iii) control conditions. Metal was added to the control medium as ZnSO_4_; Fe-EDTA; MnSO_4_; CoCl_2_; CuSO_4_; NiCl_2_ or CdCl_2_. After 3 days, the following organs were collected: (i) 3–4 cm fragment of the apical part of the root; (ii) 3–4 cm fragment of the basal part of the root; (iii) leaves (2nd and 3rd leaf counting from the base of the stem) without petioles and major midribs. The material was pooled from a total of 10 plants, frozen in the liquid nitrogen, and stored at −80°C.

To compare the *NtNRAMP3* expression between leaves of tobacco exposed to a long-term treatment of elevated Zn, 3-week-old tobacco WT seedlings were grown on a liquid control medium for 18 days, then for 3 weeks at 10, 50, and 200 μM Zn. Next, all leaves (without petioles and midvein) were removed from the stem and collected in groups of two (e.g., 1st and 2nd leaves counting from the base of the stem were the 1st pair; 3rd and 4th−2nd pair, etc.), frozen in the liquid nitrogen and stored in −80°C. The material was pooled from a total of 8 plants.

To examine the tissue-specific *NtNRAMP3* expression patterns, transgenic plants expressing *pMDC163::promNtNRAMP3::GUS* were subjected to two different treatments. In the first experiment, three-week-old plants were transferred from agar plates to hydroponics and grown under control conditions for 7 days. Whole seedlings were used to determine GUS activity. In the second experiment, 5-week-old plants grown at control conditions (3 weeks on agar plates and 2 weeks on hydroponics) were exposed to 200 μM Zn for 3 days. In the end, the second leaf (counting from the base of the stem) was collected for GUS assay. Wild-type tobacco plants were used as a negative control.

### Cloning and Generation of Constructs

A partial genomic sequence of *NtNRAMP3* (previously annotated as *NtNRAMP3-like*) was identified, and *NtNRAMP3* was reported as a candidate gene encoding metal transporter potentially involved in zinc transport in tobacco leaves (Papierniak et al., 2018). In brief, known nucleotide *NRAMP* sequences from *A. thaliana* were blasted against the *N. tabacum* genome (Sierro et al., 2013, 2014), which is deposited in GenBank, using NCBI (National Center for Biotechnology Information) BLASTn program (http://www.ncbi.nlm.nih.gov/blast). After screening with the FGENESH+ tool (SoftBerry, Mount Kisco, NY, United States), the full-length *NtNRAMP3* putative sequence was identified within the scaffold AWOK01S026429.

The full-length open reading frame (ORF) of *NtNRAMP3* (containing STOP codon or not) was amplified from cDNA transcribed from total RNA using appropriate primers (Table 1) by PCR with Phusion HF polymerase (Thermo Scientific). Then, the full-length *NtNRAMP3* sequence was cloned into the pENTR/D-TOPO vector using the pENTR Directional TOPO Cloning Kit (Invitrogen). Finally, two constructs were obtained—*pENTR/D-TOPO::NtNRAMP3* and *pENTR/D-TOPO::NtNRAMP3-STOP* (STOP codon was included).

**Table 1:** Sequences of primers used in the study.

**Primer name**	**Primer sequence**	**Product length**
**Primers for expression analysis**		
6429_1F	5' AGTTCATATCATCGGAGTCG 3'	222 bp
6429_2R	5' TGAACAAGTAGCCCAATAGCC 3'	
NtPP2A_F	5' GCACATTCATTCAGTTTGAACC 3'	142 bp
NtPP2A_R	5' GTAGCATATAAAGCAGTCAGC 3'	
**Primers for the full-length** ***NtNRAMP3*** **cDNA amplification**		
pENTR_NtNRAMP3_START	5' CACCATGCCTCCACACGATGAC 3'	1539 or 1542 bp
pENTR_NtNRAMP3_STOP	5' TCAATTCTCTATGCTGGTGATACT 3'	(including STOP codon)
pENTR_NtNRAMP3_END	5' ATTCTCTATGCTGGTGATACTCTT 3'	
**Primers for promoter** ***NtNRAMP3*** **amplification**		
promNtNRAMP3_for	5' AGAATCTGCGAGCATCTCAAAGGAATCT 3'	1684 bp
promNtNRAMP3_rev	5' TTAGAAGAGAAATCTGTAAAGAGGATATTAGCG 3'	

To prepare the construct for subcellular localization, *pENTR/D-TOPO::NtNRAMP3* was recombined with the pMDC43 vector (purchased from Arabidopsis Biological Resource Center, https://abrc.osu.edu/) using LR reaction (Gateway Technology, Invitrogen). The resulting plasmid *pMDC43::GFP::NtNRAMP3* contained GFP linked at the N-terminus of NtNRAMP3. The fusion protein was expressed under 35S promoter control.

For yeast growth assay the vectors *pAG426GAL::NtNRAMP3* were generated by LR recombination between *pENTR/D-TOPO::NtNRAMP3* and pAG426GAL-ccdB-EGFP plasmid.

For a generation of the construct for GUS analysis, a 1,684 bp promoter region located upstream from the translation initiation codon of *NtNRAMP3* was amplified from genomic DNA using appropriate primers (Table 1) by PCR with Phusion HF polymerase (Thermo Scientific) and inserted into the pENTR/D-TOPO vector using pENTR Directional TOPO Cloning Kit (Invitrogen). Then, the LR reaction was used to obtain the *pMDC163::promNtNRAMP3::GUS* construct.

### Bioinformatic Analysis

The nucleotide sequence of *NtNRAMP3* was translated to a protein sequence with the ExPASy translate tool (https://web.expasy.org/translate/). Then, to view predicted transmembrane domains of NtNRAMP3, the web-based software Protter (http://wlab.ethz.ch/protter/start/) that gathers protein features from various annotation sources, such as Uniprot, was used.

The NtNRAMP3 amino acid sequence was aligned to several chosen NRAMP amino acid sequences from other plant species (*Arabidopsis thaliana, Solanum lycopersicum, Zea mays, Theobroma caccoa*, and *Nicotiana*) identified in ARAMEMNON, Solgenomics, MaizeSequence, Phytozome, and NCBI, using Clustal Omega (https://www.ebi.ac.uk/Tools/msa/clustalo/). The phylogenetic analyses were conducted with the MEGA X program (https://www.megasoftware.net) (Kumar et al., 2018) using the maximum likelihood method with 1000 bootstrap replicates. The prediction of membrane-spanning regions was performed with Phobius software (https://phobius.sbc.su.se/) (Käll et al., 2004).

All primers used in this study were designed with the OligoAnalyzer tool (https://www.idtdna.com/pages/tools/oligoanalyzer) based on the sequences of *NtNRAMP3* (primers for expression analysis and *NtNRAMP3* ORF amplification) and scaffold AWOK01S026429 (primers for *NtNRAMP3* promoter amplification). Primers sequences are listed in Table 1.

Regulatory elements within the *NtNRAMP3* promoter region were identified with the program PlantCARE (http://bioinformatics.psb.ugent.be/webtools/plantcare/html/).

### Generation of Transgenic Plants

The construct *pMDC163::promNtNRAMP3::GUS* was stably transformed into tobacco plants using GV3101 *A. tumefaciens*-mediated transformation protocol for tobacco leaf disks (described in Siemianowski et al., 2011). Transgenic plants were selected in the presence of hygromycin B (100 μg·ml^−1^). The T1 heterozygous lines with a segregation ratio of 3:1 (hygromycin^toler^: hygromycin^sensit^) were self-pollinated to obtain the homozygous generation (T2). Developed independent homozygous lines were tested in a preliminary histochemical GUS assay in young 4-week-old seedlings, subsequently three lines were selected for more detailed analysis.

### Quantitative RT-PCR Analysis

Total RNA extraction and quantitative real-time RT-PCR (qPCR) analysis were performed according to procedures described in Papierniak et al. (2018). In brief, Plant RNA Mini Kit (Syngen, Wrocław, Poland) was used for RNA isolation, then samples were digested with DNase I (Invitrogen, Waltham, MA, United States). The relative quantities of each transcript were calculated based on the comparative ΔCt (threshold cycle) method (Livak and Schmittgen, 2001). The *NtPP2A* (*Nicotiana tabacum Protein Phosphatase 2 A*) gene was used as an internal control, and its stability was measured in all plant tissue samples in the range of applied metal concentrations. At least three independent biological replicates were analyzed for each experiment. A minimum 2-fold change in relative gene expression level was considered significant.

### Functional Analysis of NtNRAMP3 in Yeast

In the study, two wild-type (DY1457 and BY4742) and three mutants (Δ*smf1*, Δ*zrt1*, Δ*fet3fet4*) *Saccharomyces cerevisiae* strains (Supplementary File S1) were transformed with the empty vector *pAG426GAL* or with *pAG426GAL::NtNRAMP3* construct, according to lithium acetate method protocol (Gietz and Schiestl, 2007). Complementation and sensitivity tests were performed as described previously (Papierniak-Wygladala et al., 2020). Briefly, yeast cultures grown on liquid synthetic medium (SC-URA+GLU; yeast nitrogen base, amino acids without uracil, 2% glucose, pH 5.3), after setting the optical density (OD_600_) to 0.2, were serially diluted (1.0; 0.1; 0.01 and 0.001), and then spotted on Petri dishes containing SC-URA+GAL solidified with 2% (w/v) agar and supplemented with required components (details below). Yeast growth was monitored for the next up to 10 days.

In complementation tests, for the Δ*fet3fet4* mutant, the restrictive medium was supplemented with (i) 10 or 30 μM FeCl_3_, (ii) 30 or 100 μM ferric citrate, and/or (iii) 30 or 80 μM BPDS (bathophenanthrolinedisulfonic acid). For Δ*smf1* to the medium 2.5 mM EGTA (Ethylene glycol-bis(β-aminoethyl ether)-*N, N, N*′, *N*′-tetraacetic acid) alone or with 0.1 mM MnCl_2_ (pH was adjusted to 6.0 with 50 mM MES or SC-URA+GAL medium of pH 5.3 was applied) were added. The Δ*zrt1* mutant was assayed for growth on medium containing (i) 1.0 mM EDTA (Ethylenediaminetetraacetic acid), and/or (ii) 100, 500, 600, or 700 μM ZnCl_2_. Wild-type strains transformed with the empty *pAG426GAL* vector were included as a reference control in all assays (DY1457 in experiments including Δ*fet3fet4* and BY4742 in experiments including Δ*smf1* and Δ*zrt1*). Mutant strains transformed with the empty *pAG426GAL* vector were included as a negative control in all experiments.

In sensitivity tests, the growth of DY1457 yeast transformed with *pAG426GAL::NtNRAMP3* construct was monitored on plates containing SC-URA+GAL supplemented with (i) 5, 10, 20, 50, or 75 μM CdCl_2_, (ii) 50, 100, 250, 500, or 750 μM CoCl_2_, (iii) 1.0, 1.4, 2.0, 2.2, or 2.4 mM CuSO_4_, (iv) 0.2, 3.0, 4.0, 4.5, or 5.0 mM FeCl_3_, (v) 0.2, 2.5, 5, 7.5, or 10.0 mM MnCl_2_, (vi) 0.2, 0.3, 0.4, 0.5, or 0.6 mM NiCl_2_, (vii) 0.2, 1.0, 2.5, 5.0, or 7.5 mM ZnSO_4_, and (viii) 4.0, 4.5, 5.0, 5.5, or 6.0 mM ZnSO_4_ (pH was adjusted to 4.0, 5.0, or 6.0 with 50 mM MES). DY1457 transformed with the empty *pAG426GAL* vector was included as a control in all assays.

### Subcellular Localization of NtNRAMP3

The subcellular localization of NtNRAMP3 was determined by monitoring the transient expression of GFP-NtNRAMP3 translational fusion product in tobacco leaf epidermal cells accordingly to the previously described protocol (Siemianowski et al., 2013; Papierniak et al., 2018). The GV3101 *A. tumefaciens* (C58C1, Rif^R^; pMP90, Gm^R^) transformed with *pMDC43::GFP::NtNRAMP3* were used for infiltration of 7-week-old WT tobacco leaves. To visualize the cell wall, after 24–72 h from the infiltration leaf fragments were stained with 50 μM water solution of propidium iodide (PI) for 20 min. Then, GFP (excitation: 488 nm line of argon laser; emission: 500–560 nm) and PI (excitation: 543 nm; emission: 617 nm) signals were detected in Nikon A1 confocal laser scanning microscope (Melville, NY, USA).

### Histochemical GUS Analysis

The GUS assay in young seedlings (4-week-old whole plants) was performed according to Weremczuk et al. (2020). In brief, 4-week-old whole seedlings were fixed in 90% ice-cold acetone and washed in the 50 mM phosphate buffer pH 7.0 with 0.2 % Triton X-100 with infiltration. Then, the plant material was incubated in a reaction buffer (50 mM phosphate buffer pH 7.0 with 0.2 % Triton X-100 and 2 mM X-Gluc), at 37°C in the darkness for 2.5 h. Next, they were fixed in FAA (formalin-acetic acid-alcohol) for 30 min, dehydrated in increasing ethanol concentrations, and stored in 70% ethanol. To visualize expression sites of *NtNRAMP3* within leaves of 5.5-week-old tobacco, we used the method described in Weremczuk et al. (2020). In brief, the determination of GUS activity in the leaves of 5.5-week-old tobacco was carried out on cross-sections of 130 μm thickness cut on a Vibratome (Leica VT1000S, Heidelberg, Germany). They were used for histochemical GUS staining, subsequently fixed in FAA for 30 min, dehydrated in increasing ethanol concentrations, and stored in 70% ethanol. Observations were performed with an OPTA-TECH microscope.

## Results

### Characterization of NtNRAMP3 and Phylogenetic Analysis of Plant NRAMP Proteins

Earlier studies showed that the partial sequence of *NtNRAMP3-like* first identified in tobacco within the scaffold AWOK01S026429 was upregulated in the leaves by 200 μM Zn (Papierniak et al., 2018). Here, this sequence has been cloned. The full-length cDNA is comprised of 1,542 bp and encodes a protein of 514 amino acids (**Figure 2**; FGENESH+). Analyses of the nucleotide and amino acid sequences provided evidence that the identified sequence is *NtNRAMP3*.

**Figure 1:**
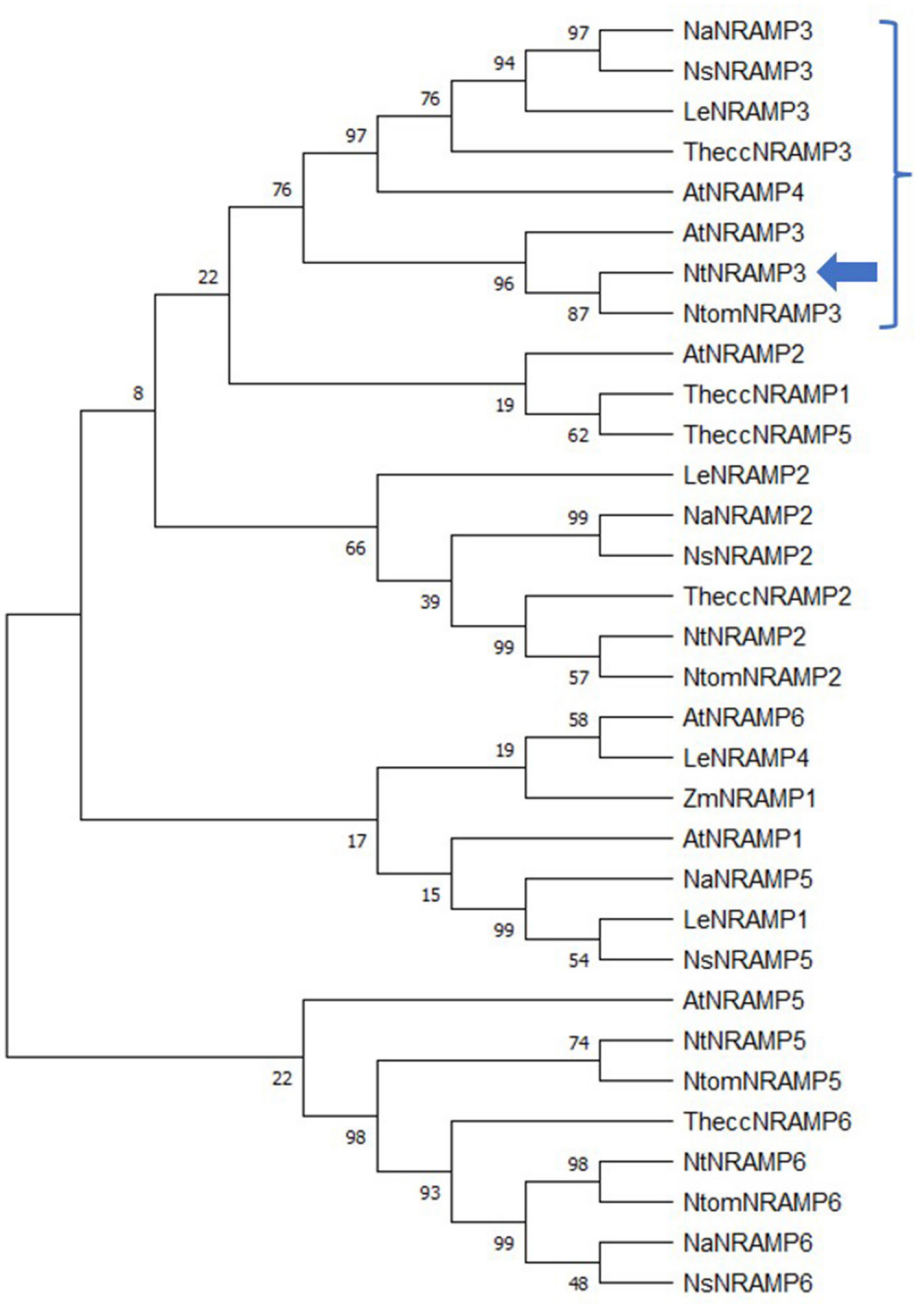
A phylogenetic tree of the natural resistance-associated macrophage protein (NRAMP) transporters from *Arabidopsis thaliana, Solanum lycopersicum, Zea mays, Theobroma cacao*, and *Nicotiana*. Amino acid sequences of the 32 NRAMP proteins were identified in the ARAMEMNON (*A.thaliana*), Solgenomics (*S. lycopersicum*), MaizeSequence (*Z. mays*), Phytozome (*T. cacao*), and NCBI (*Nicotiana*) databases. The phylogenetic tree was constructed with the MEGA X software using Neighbor-Joining method. The length of branches corresponds to the degree of divergence. Numbers in the figure represent bootstrap values (1,000 replicates). Accession numbers for all proteins applied in the analysis: (i) *A. thaliana*: AtNRAMP1, AT1G80830; AtNRAMP2, AT1G47240; AtNRAMP3, AT2G23150; AtNRAMP4, AT5G67330; AtNRAMP5, AT4G18790; AtNRAMP6, AT1G15960; (ii) *S. lycopersicum*: LeNRAMP1, Solyc11g018530; LeNRAMP2, Solyc04g078250; LeNRAMP3, Solyc02g092800; LeNRAMP4, Solyc03g116900; (iii) *Z. mays*: ZmNRAMP1, GRMZM2G178190; (iv) *T. cacao*: TheccNRAMP1, Thecc1EG035168; TheccNRAMP2, Thecc1EG034751; TheccNRAMP3, Thecc1EG000729; TheccNRAMP5, Thecc1EG035174; TheccNRAMP6, Thecc1EG027424; (v) *Nicotiana attenuata*: NaNRAMP2, XP_019262545; NaNRAMP3, XP_019228309; NaNRAMP5, XP_019245559; NaNRAMP6, XP_019243869; (vi) *Nicotiana sylvestris*: NsNRAMP2, XP_009760309; NsNRAMP3, XP_009796782; NsNRAMP5, XP_009783885; NsNRAMP6, XP_009774026; (vii) *Nicotiana tabacum*: NtNRAMP2, XP_016477061; NtNRAMP3, NP_001312209 (marked with blue arrow); NtNRAMP5, XP_016434268; NtNRAMP6, XP_016480878; (viii) *Nicotiana tomentosiformis*: NtomNRAMP2, XP_009620156; NtomNRAMP3, XP_009616361; NtomNRAMP5, XP_009620069; NtomNRAMP6, XP_009594426. A bracket-marked branch of the phylogenetic tree indicates NRAMP proteins which are the most closely related to NtNRAMP3.

First, phylogenetic analysis that included 32 NRAMP proteins from *A. thaliana, S. lycopersicum, T. cacao, Z. mays*, and *Nicotiana* species (*N. attenuata, N. sylvestris, N. tomentosiformis, N. tabacum*) showed that the newly identified protein was located on the same branch as other NRAMP3 proteins, with the closest relationship between NtomNRAMP3 and AtNRAMP3 (Figure 1). In general, the examined proteins were classified into four groups. Among them, NtNRAMP3 formed a separate clade with NaNRAMP3, NsNRAMP3, NtomNRAMP3, TheccNRAMP3, LeNRAMP3, AtNRAMP3, and AtNRAMP4, which was distinct from other NRAMPs (Figure 1, marked with bracket). Furthermore, among all NRAMP proteins included in the study, NtNRAMP3 was the most similar to NtomNRAMP3 (99.81%), and the highest identity of the amino acid sequences was observed within *Nicotiana* (98.82% and 98.23% between NtNRAMP3 and NaNRAMP3 and NsNRAMP3, respectively, Table 2). On the other hand, the lowest identity within this group was observed between NtNRAMP3 and AtNRAMP4 (73.81%).

**Table 2:** Amino acids sequence identity between selected NRAMP proteins.

	**NtomNRAMP3**	**NaNRAMP3**	**NsNRAMP3**	**LeNRAMP3**	**TheccNRAMP3**	**AtNRAMP3**	**AtNRAMP2**	**AtNRAMP4**	**AtNRAMP1**
NtNRAMP3	99.81	98.82	98.23	91.14	78.94	77.18	73.90	73.81	38.78
NtomNRAMP3		98.62	98.04	91.14	78.94	77.18	73.90	73.81	38.78
NaNRAMP3			98.23	90.93	79.56	77.00	74.70	74.05	39.51
NsNRAMP3				90.14	79.17	77.00	74.30	74.05	39.09
LeNRAMP3					76.98	75.60	73.90	72.46	39.14
TheccNRAMP3						80.20	76.00	74.60	40.12
AtNRAMP3							75.15	76.74	38.37
AtNRAMP2								70.32	37.80
AtNRAMP4									38.46

*Abbreviations: At, Arabidopsis thaliana; Le, Solanum lycopersicum; Na, Nicotiana attenuata; Ns, Nicotiana sylvestris; Nt, Nicotiana tabacum; Ntom, Nicotiana tomentosiformis; Thecc, Theobroma cacao. All data is shown in percent [%]. Gray cells indicate repeated data*.

**Figure 2:**
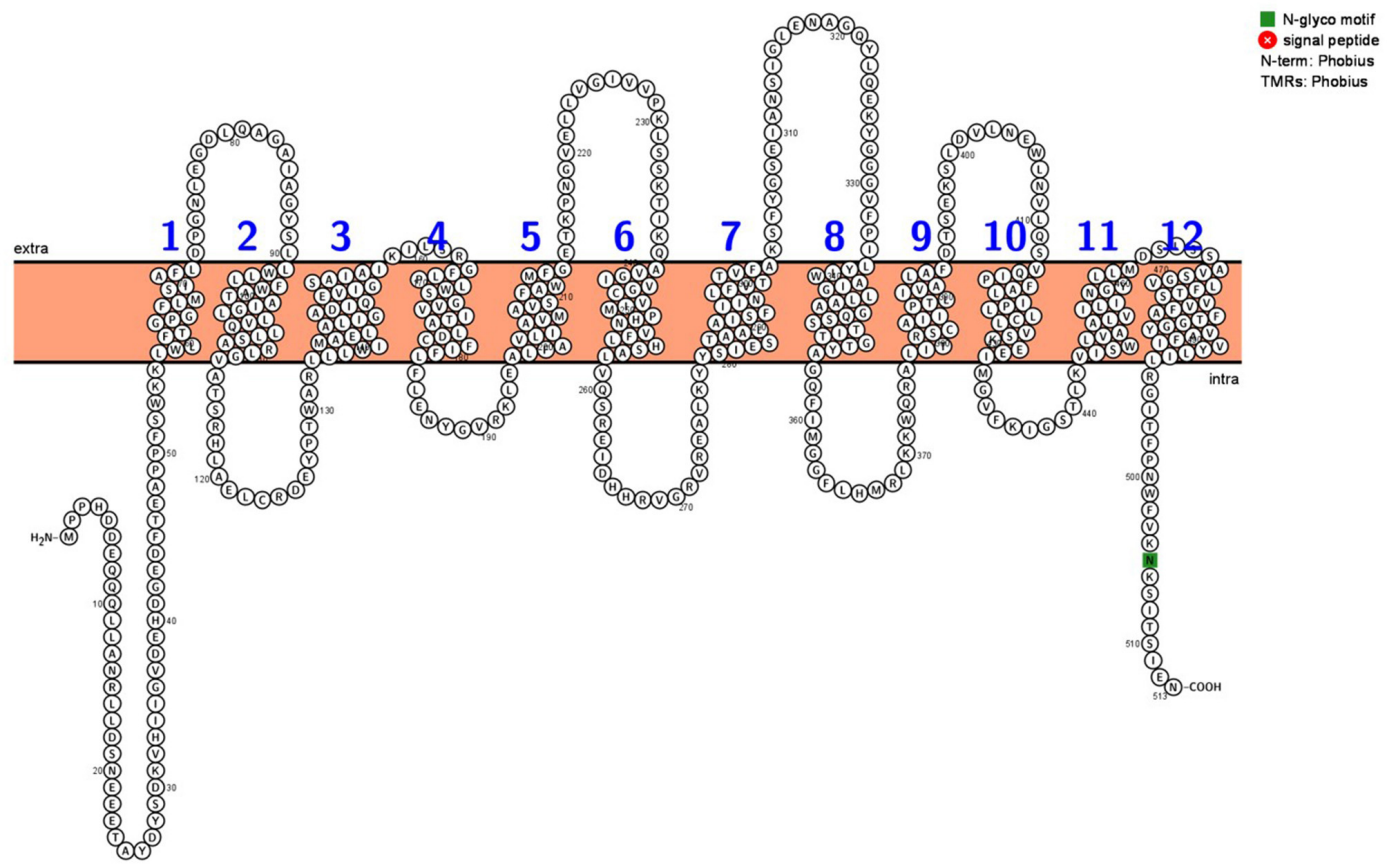
Predicted transmembrane topology map of NtNRAMP3. The topology map was generated using the web-based Protter software (Omasits et al., 2014). NtNRAMP3 protein structure is comprised of 12 transmembrane regions and one N-glycosylation motif at amino acid position 505 (in green). N- and C- ends of NtNRAMP3 protein are located intracellularly.

Like many other NRAMP proteins, NtNRAMP3 consists of 12 transmembrane domains (TMDs) and has both N- and C-termini located intracellularly (Phobius, Käll et al., 2004; Protter, Omasits et al., 2014) (Figure 2; Supplementary File S2). *In silico* analysis based on Phobius and Protter suggested also that NtNRAMP3 has one N-glycosylation motif of amino acid position 505 located in the C-terminus.

Second, multiple amino acid sequence alignment showed high conservation between the NtNRAMP3 and examined NRAMP proteins (Supplementary File S2). NtNRAMP3 contains key sequences considered typical for NRAMP proteins. The consensus transport motif (CTM) [GQSSTIT(/A)G(/D)TYAGQY(/F)V(/I)MQ(/G/E)GFLD(/H/N)], which is the signature sequence of NRAMP family, was present in all examined proteins. Moreover, three highly conserved histidine residues (one within the region between II and III TMD and two within VI TMD) were identified.

Histidine residues occur at different locations in the NRAMP protein sequences. The location of some is highly conserved for all examined NRAMP proteins (as between II and III TMD and within VI TMD). Others are distributed in various locations within the sequence, however, the same for certain groups of plants. Thus, in the structure of NtNRAMP3 together with NtomNRAMP3, NaNRAMP3, NsNRAMP3, and LeNRAMP3, an additional histidine was present within CTM. Furthermore, additional histidine residues, H4 and H40, were present in the N-terminus of NtNRAMP3, and also within NtomNRAMP3, NaNRAMP3, and NsNRAMP3 (Supplementary File S2).

### *NtNRAMP3* Encodes a Plasma Membrane Protein

**Figure 3:**
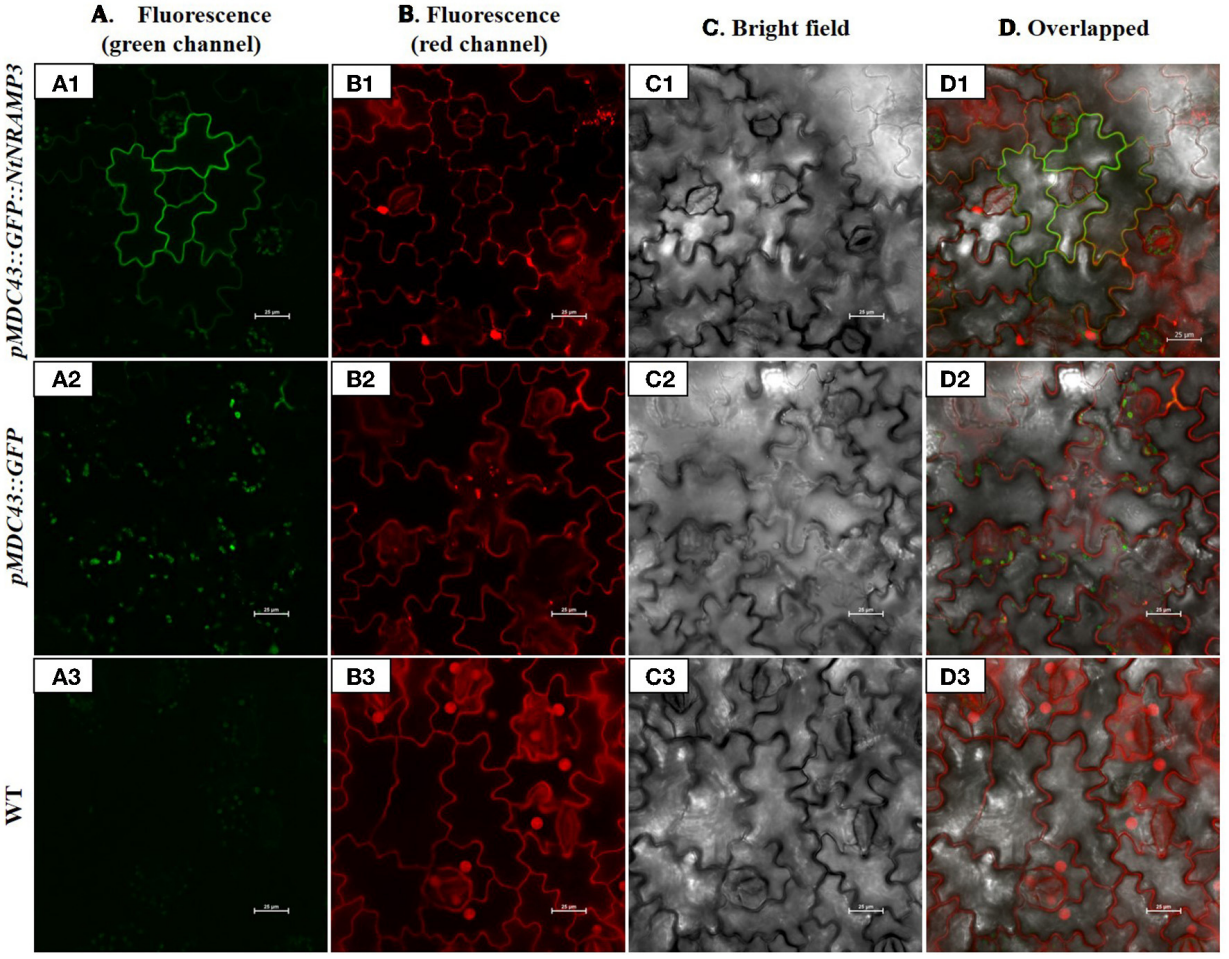
Subcellular localization of NtNRAMP3 in the epidermal cells of tobacco leaves. *A. tumefaciens* GV3101 strains containing the *pMDC43::GFP* [control empty vector **(A2,B2,C2,D2)**] or *pMDC43::GFP::NtNRAMP3* **(A1,B1,C1,D1)** were used for transient expression in *N. tabacum* leaves. After 1–4 days from infiltration leaves were observed under a confocal microscope. **(A)** GFP fluorescence (green channel); **(B)** PI (propidium iodide) signal in the cell wall (red channel); **(C)** bright-field images; **(D)** merged images. WT tobacco plants **(A3,B3,C3,D3)** were used as a negative control for the GFP signal. The white bar represents 25 μm.

To determine the subcellular localization of NtNRAMP3 protein, the *pMDC43::GFP::NtNRAMP3* construct was transiently expressed in tobacco leaf epidermal cells (Figure 3). The green fluorescence was present along the contours of the strongly folded cell walls of the lower epidermal cells (Figure 3A1) and overlapped with the red signal coming from the cell walls stained with propidium iodide (Figures 3B1,D1). Since the plasma membrane sticks to the primary cell wall, it is not distinguished by confocal light microscopy. Thus, the detected colocalization of both green and red signals indicates that NtNRAMP3 is localized in the plasma membrane. It is also known that the central vacuole does not enter the narrow projections of the epidermal cell, so the lack of a green signal at their bases additionally confirms the location of the tested protein in the plasma membrane (Siemianowski et al., 2013; Pighin et al., 2014; Barabasz et al., 2019). Autofluorescence was extremely low (Figures 3A3,D3). The green fluorescence of GFP alone was observed only in small circular structures within the cells (Figures 3A2,D2).

### Substrate Specificity of NtNRAMP3

Natural resistance-associated macrophage proteins are typically involved in the transport of several metals, including Fe, Mn, Ni, Co, Cu, Zn, and Cd (Gunshin et al., 1997; Thomine et al., 2000; Mizuno et al., 2005; Wang et al., 2019). Here, to identify substrates for NtNRAMP3, we applied two types of yeast growth assay—sensitivity tests (comparison of the growth of WT yeast transformed with either empty *pAG426GAL* vector or the construct *pAG426GAL::NtNRAMP3*) and complementation tests (comparison of the growth of mutant yeast transformed with either empty *pAG426GAL* vector or the construct *pAG426GAL::NtNRAMP3*; as the reference WT yeast transformed with the empty vector was used). Accordingly, *NtNRAMP3* expression was tested in the selected mutants (Δ*fet3fet4*, Δ*smf1*, or Δ*zrt1*) and WT (DY1457 or BY4742) yeast strains.

**Figure 4:**
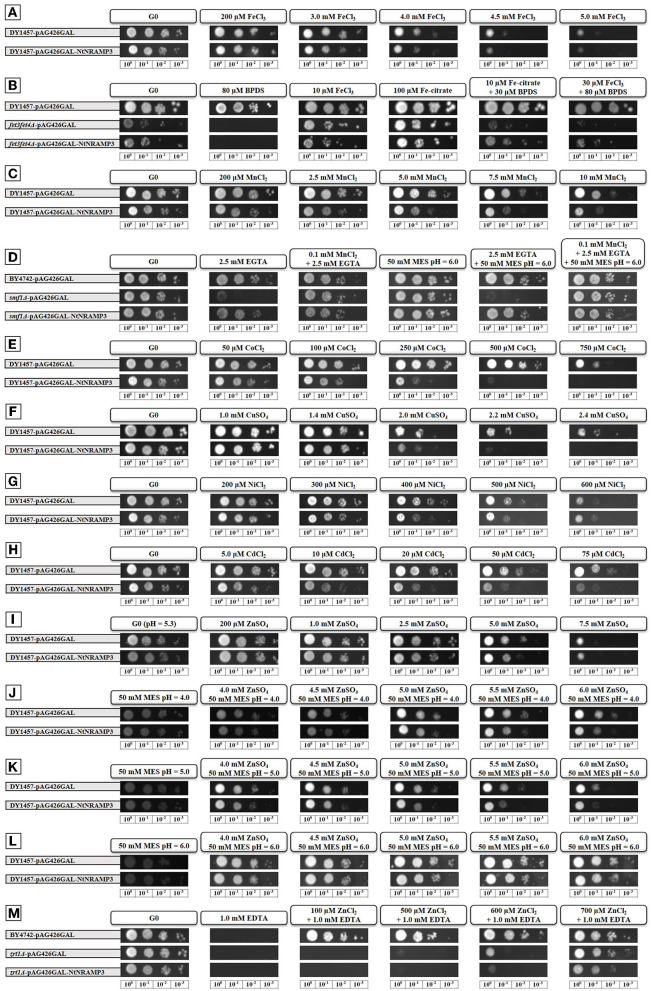
*NtNRAMP3* expression in selected yeast strains. *Saccharomyces cerevisiae* strains (i) wild-type (WT; DY1457 or BY4742) and (ii) deletion mutants Δ*fet3fet4* (defective in Fe transport into the cell), Δ*smf1* (defective in Mn transport into the cell) or Δ*zrt1* (defective in Zn transport into the cell) were transformed with the empty vector *pAG426GAL* or with the construct *pAG426GAL::NtNRAMP3*. The transformed cells were adjusted to OD_600_ = 0.2, then a series of dilutions (1, 0.1, 0.01, and 0.001) were prepared. Yeast cultures were spotted (5 μl aliquotes) on SC-URA+GAL plates supplemented with required components and incubated at 30°C for up to 10 days. *Sensitivity tests*: Transformed DY1457 cells on SC-URA+GAL plates supplemented with: **(A)** 200 μM, 3.0, 4.0, 4.5, or 5.0 mM FeCl_3_; **(C)** 200 μM, 2.5, 5.0, 7.5, or 10.0 mM MnCl_2_; **(E)** 50, 100, 250, 500, or 750 μM CoCl_2_; **(F)** 1.0, 1.4, 2.0, 2.2, or 2.4 mM CuSO_4_; **(G)** 200, 300, 400, 500, or 600 μM NiCl_2_; **(H)** 5, 10, 20, 50, or 75 μM CdCl_2_; **(I)** 200 μM, 1.0, 2.5, 5.0, or 7.5 mM ZnSO_4_; **(J)** 4.0, 4.5, 5.0, 5.5, or 6.0 mM ZnSO_4_ (pH was adjusted to 4.0 with 50 mM MES); **(K)** 4.0, 4.5, 5.0, 5.5, or 6.0 mM ZnSO_4_ (pH was adjusted to 5.0 with 50 mM MES) or **(L)** 4.0, 4.5, 5.0, 5.5, or 6.0 mM ZnSO_4_ (pH was adjusted to 6.0 with 50 mM MES). DY1457 transformed with the empty vector *pAG426GAL* was used as a control. The same control plate is depicted in **(A,E,F,G)**—the experiments were done simultaneously. **(A,E,G)** Depict data from day 3, **(F)**—data from day 5. The same control plate is depicted in **(C,H)**—the experiments were done simultaneously (data from day 3). *Complementation tests*: **(B)** Transformed *fet3fet4*Δ cells on SC-URA+GAL plates supplemented with 80 μM BPDS; 10 μM FeCl_3_; 100 μM Fe-citrate; 10 μM Fe-citrate + 30 μM BPDS; 30 μM FeCl_3_ + 80 μM BPDS. **(D)** Transformed *smf1*Δ cells on SC-URA+GAL plates supplemented with 2.5 mM EGTA; 0.1 mM MnCl_2_ + 2.5 mM EGTA; 50 mM MES pH = 6.0; 2.5 mM EGTA + 50 mM MES pH = 6.0; 0.1 mM MnCl_2_ + 2.5 mM EGTA + 50 mM MES pH = 6.0. **(M)** Transformed *zrt1*Δ cells on SC-URA+GAL plates supplemented with 1 mM EDTA; 100 μM ZnCl_2_ + 1 mM EDTA; 500 μM ZnCl_2_ + 1 mM EDTA; 600 μM ZnCl_2_ + 1 mM EDTA; 700 μM ZnCl_2_ + 1 mM EDTA. DY1457 (for *fet3fet4*Δ) or BY4742 (for *smf1*Δ and *zrt1*Δ) transformed with the empty vector *pAG426GAL* was used as a control.

First, the sensitivity of strain DY1457 (WT) expressing *pAG426GAL* or *pAG426GAL::NtNRAMP3* to various concentrations of Fe, Mn, Co, Cu, Ni, Cd, and Zn was compared. Expression of *NtNRAMP3* led to inhibition of yeast growth in the presence of high concentrations of Fe (Figure 4A), Mn (Figure 4C), Co (Figure 4E), Cu (Figure 4F), Ni (Figure 4G), or Cd (Figure 4H). However, expression of *NtNRAMP3* in strain DY1457 increased yeast sensitivity to Zn only in the presence of a strictly defined concentration—around 5.0 mM (Figure 4I). To learn more, the experiments were extended by the use of three pH values (4.0, 5.0, and 6.0 adjusted with 50 mM MES; the pH of the SC-URA+GAL medium was 5.3) and five concentrations of Zn (4.0; 4.5; 5.0; 5.5; 6.0 mM). Likewise, *NtNRAMP3*-expressing yeast grew slower with the smallest difference to the control at 4.0 mM Zn applied at all tested pH values (Figures 4J–L).

Next, yeast mutants defective in Fe and Mn uptake were used. The expression of *NtNRAMP3* restored the growth of Δ*fet3fet4* (Figure 4B) and Δ*smf1* (Figure 4D) under Fe- or Mn-limited conditions, respectively. In contrast, expression of *NtNRAMP3* did not restore the growth of the Δ*zrt1* mutant under Zn-limited conditions induced with a strong chelator EDTA (Figure 4M), and the growth of Δ*zrt1* yeast carrying either the empty *pAG426GAL* vector or the construct (*pAG426GAL::NtNRAMP3*) was severely restricted.

These results indicate that NtNRAMP3 is an influx transporter for Fe, Mn, Co, Cu, Ni, and also toxic Cd. It also seems to transport Zn, but probably only under very specific environmental conditions/medium composition.

### Developmental Regulation of *NtNRAMP3* Expression

**Figure 5:**
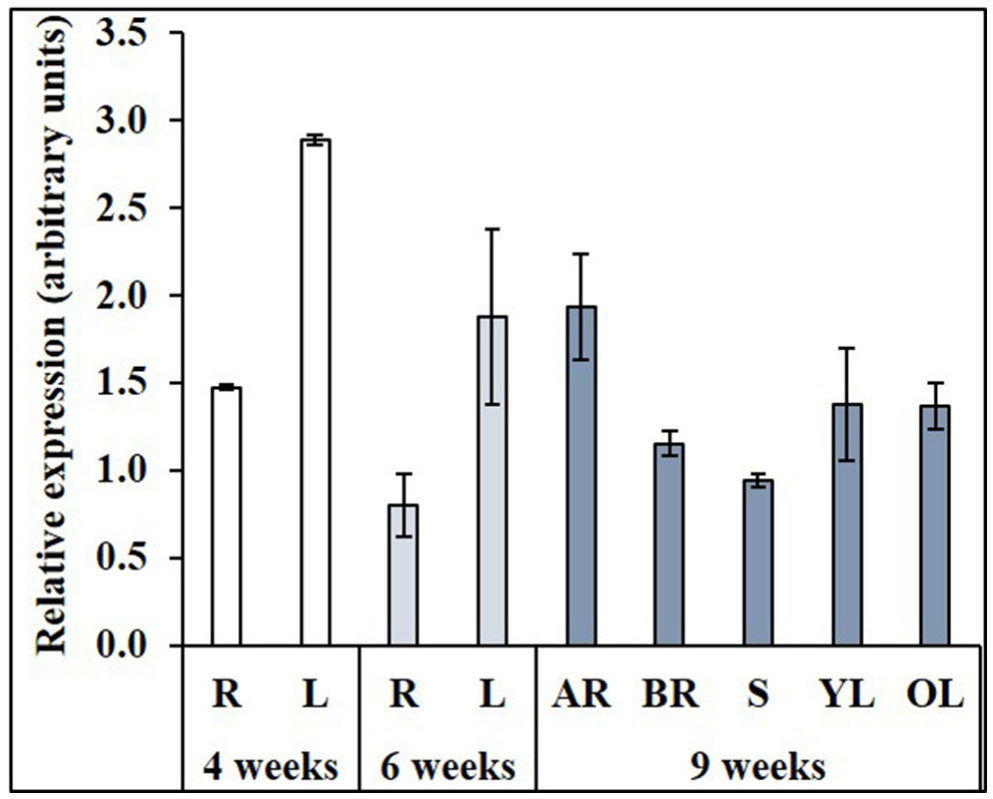
Expression of *NtNRAMP3* during development. WT tobacco plants were grown at the control conditions ($\frac{1}{4}$ Knop's medium) for up to 9 weeks. At three selected developmental stages *NtNRAMP3* transcript level was monitored by RT-qPCR and normalized to the *NtPP2A* expression level. Expression of *NtNRAMP3* was determined in (i) roots (R) and leaves (L) of 4-week-old plants; (ii) roots (R) and leaves (L) of 6-week-old plants; (iii) apical (AR) and basal fragments of roots, stem (S), young (YL) and old (OL) leaves of 9-week-old plants. Values correspond to mean ± *SD* (*n* ≥ 3); those with a ratio greater than 2 are considered significantly different (Jain et al., 2013).

To determine the role of *NtNRAMP3* in tobacco, the level of its expression in organs during vegetative development under control conditions was analyzed (Figure 5). In young 4-week-old and also in 6-week-old plants *NtNRAMP3* was preferentially expressed in the developing leaves, and its expression level was 2- to 3-fold higher in leaves compared with roots. The pattern changed in older, 9-week-old plants. Studies carried out on the apical and basal parts of the root, the stem, and young and older leaves did not show any significant differences. In general, however, the level was moderately lower compared with high expression in the leaves of younger plants.

### Metal Status-Dependent Expression of *NtNRAMP3*

Knowing that NtNRAMP3 mediates the transport of Fe, Mn, Co, Ni, Cu, and Cd, subsequent experiments were performed to define how *NtNRAMP3* is regulated by the status of these metals. The main question was whether it is actively involved in the plant's response to excess metals or the supply of metals upon their deficiency. The appearance of plants after exposure to the tested metals is illustrated in Supplementary File S3. Organs, such as apical and basal parts of the roots and leaves, were included in the study. The stability of the reference *NtPP2A* gene is shown in Supplementary File S4.

**Figure 6:**
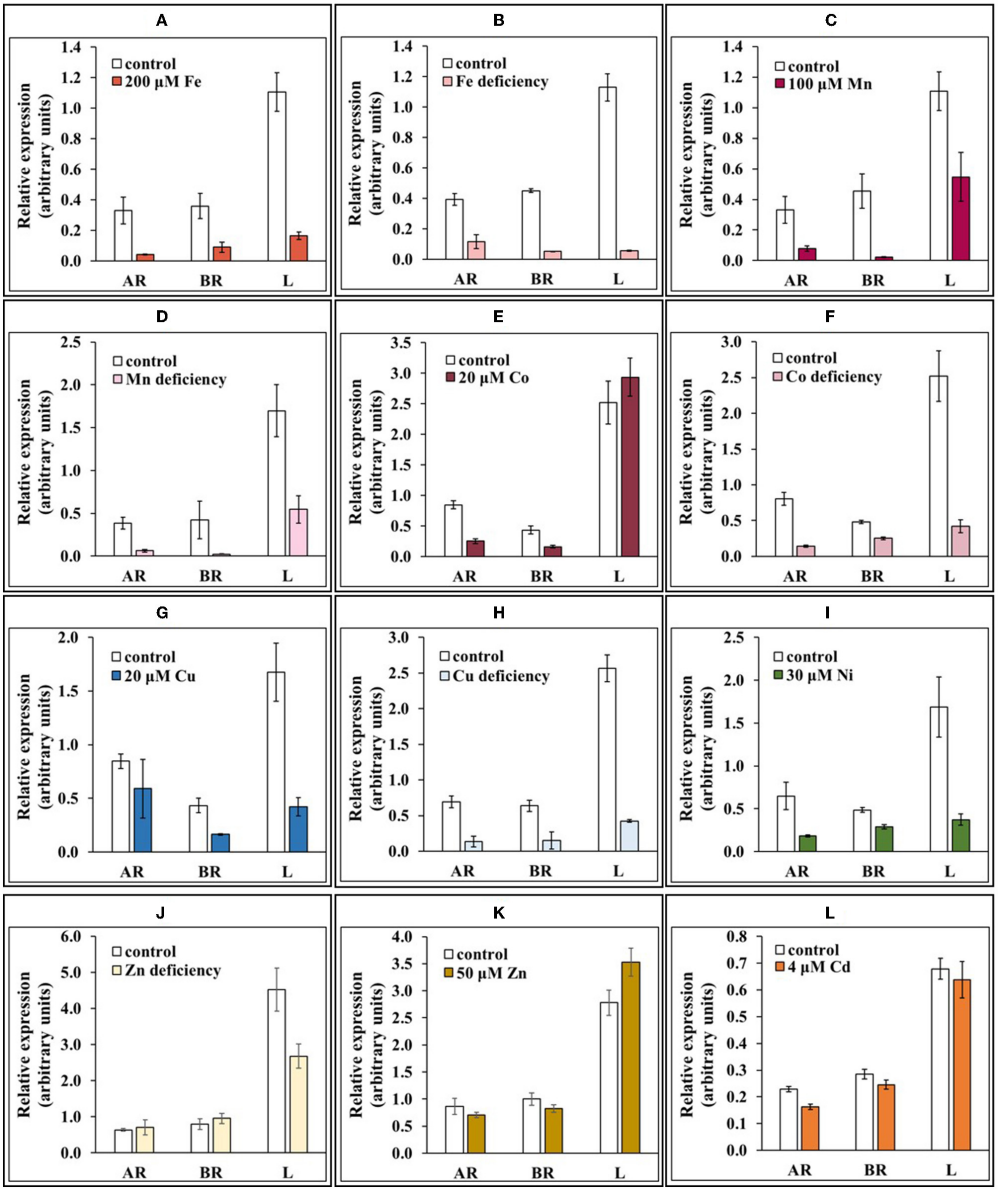
Expression of *NtNRAMP3* under different metal regimes. **(A–I,L)** Wild-type (WT) tobacco plants were grown at control conditions ($\frac{1}{4}$ Knop's medium) for 5 weeks and then were subjected to the different metal regimes for the next 4 days: **(A)** 200 μM Fe; **(B)** Fe deficiency; **(C)** 100 μM Mn; **(D)** Mn deficiency; **(E)** 20 μM Co; **(F)** Co deficiency; **(G)** 20 μM Cu; **(H)** Cu deficiency; **(I)** 30 μM Ni; **(J)** Zn deficiency; **(K)** 50 μM Zn; **(L)** 4 μM Cd. In parallel, WT tobacco plants were grown at control conditions. The expression of *NtNRAMP3* was determined in apical (AR) and basal (BR) segments of the roots, and in leaves **(L)**. *NtNRAMP3* transcript level was monitored by RT-qPCR and normalized to the *NtPP2A* expression level. Values correspond to mean ± *SD* (*n* ≥ 3); those with a ratio >2 are considered significantly different (Jain et al., 2013).

With some exceptions, the highest level of expression of *NtNRAMP3* was detected under control conditions. In experiments with the deficiency of Fe, Mn, Co, or Cu, *NtNRAMP3* expression decreased significantly in all tested organs compared with control conditions (Figures 6B,D,F,H). Considering Zn deficiency, *NtNRAMP3* was lower only in the leaves (Figure 6J). Importantly, the transcript level also decreased after plants were exposed to high concentrations of metals (200 μM Fe or 100 μM Mn or 20 μM Co or 20 μM Cu or 30 μM Ni), excluding leaves and apical root parts after exposure to Co and Cu, respectively (Figures 6A,C,E,G,I). Also, no changes in the expression level were found after exposure to 50 μM Zn (Figure 6K), as well as in the presence of 4 μM Cd (Figure 6L).

**Figure 7:**
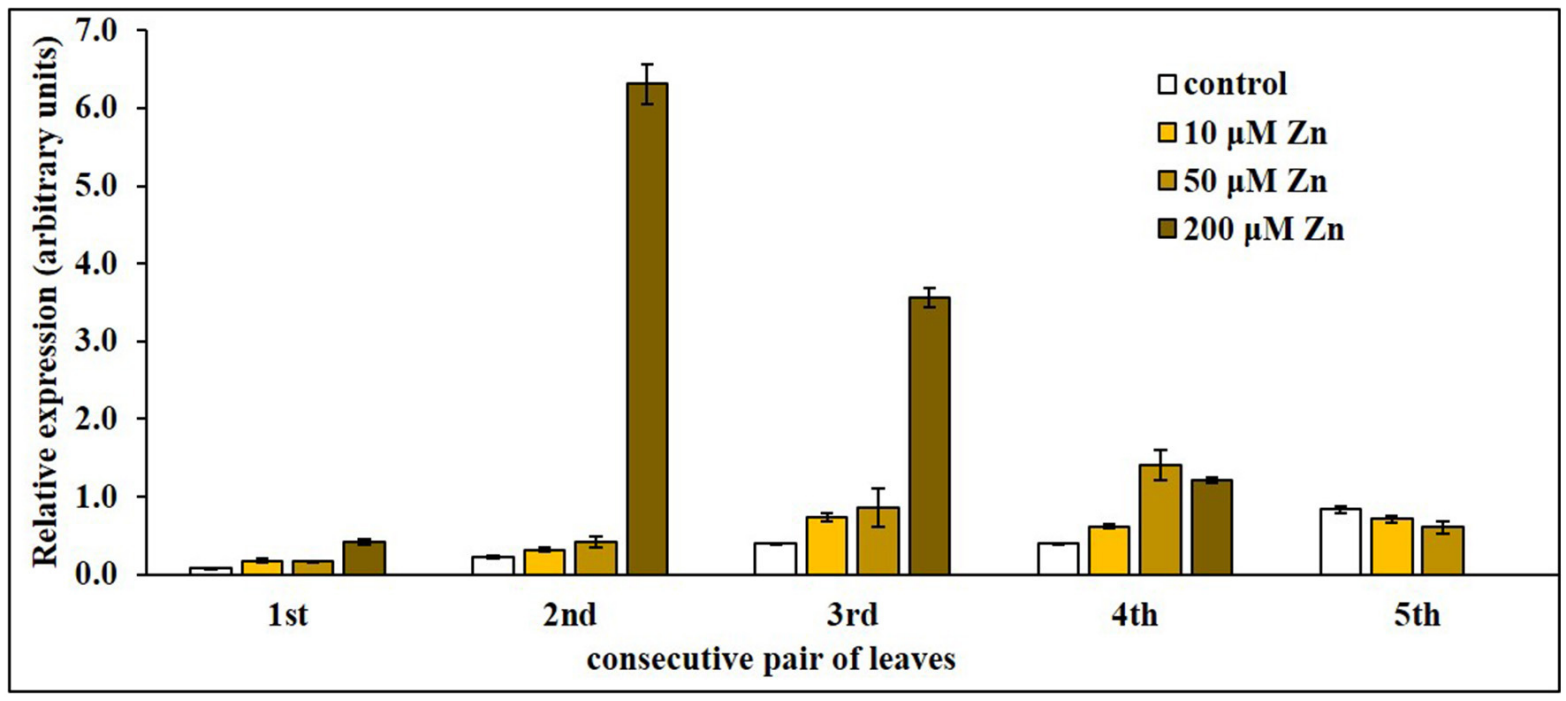
*NtNRAMP3* in consecutive pairs of leaves of plants grown under different zinc regimes. 5.5-week-old wild-type (WT) tobacco plants were transferred for the next 3 weeks to control medium ($\frac{1}{4}$ Knop's) supplemented with: (i) 10 μM Zn; (ii) 50 μM Zn; (iii) 200 μM Zn. In parallel, WT tobacco plants were grown at control conditions. Expression of *NtNRAMP3* was determined in consecutive pairs of leaves (numbered from 1 to 5; 1st and 2nd leaves counting from the base of the stem were the 1st pair, etc.). *NtNRAMP3* transcript level was monitored by RT-qPCR and normalized to the *NtPP2A* expression level. Values correspond to mean ± *SD* (*n* ≥ 3); those with a ratio >2 are considered significantly different (Jain et al., 2013).

A previous study showed that the expression of *NtNRAMP3* in tobacco leaves went up in response to up to 4-day exposure to very high (200 μM) Zn, suggesting *NtNRAMP3* involvement in plants' response to toxic zinc levels (Papierniak et al., 2018). To initially verify this possible function, a more detailed examination of the transcript level in consecutive pairs of leaves of long-term exposed tobacco plants to elevated concentrations of Zn was performed. Two key pieces of information were obtained in this experiment. The expression of *NtNRAMP3* was strongly induced by high Zn concentration, however, in a manner dependent on the age/position on the stem, but it was not regulated in the youngest leaves (5th pair) (Figure 7). The highest upregulation resulted from exposure to 200 μM Zn in the 2nd and 3rd pair of leaves. The 21-day exposure to 50 μM Zn also resulted in enhanced expression, however, to a much lesser extent.

Taken together, our results indicate a general role of *NtNRAMP3* in the regulation of the balance of several metals in plants grown under control conditions, with a possible specific role at exposure to extreme Zn-imposed stresses.

### The Activity of the *NtNRAMP3* Promoter Is Limited to Specific Tissues

#### Regulatory Sequences Identified Within the NtNRAMP3 Promoter Sequence

The sequence of the *NtNRAMP3* promoter region (−1,684 bp upstream to ATG) is shown in Supplementary File S5. Within its sequence, two *cis*-regulatory elements potentially involved in a plant's metal stress response, MRE1 (metal-responsive element 1, Li et al., 2013) and IDE2 (iron deficiency response element 2, Ogo et al., 2008), were identified. However, the MRE1 sequence in the *NtNRAMP3* promoter was incomplete. The presence of the TGCACC sequence was determined, thus compared with the original sequence, TGC(A/G)C(T/G/C/A)C (Li et al., 2013), it was lacking the last nucleotide. The *NtNRAMP3* promoter also contained *cis*-regulatory elements known to determine the plant's response to phytohormones (ABRE, CGTCA-motif), light (BOX4, G-box, GATA-motif, TCCC-motif, chs-CMA1a) and abiotic stresses (ARE) (Fink et al., 1988; Lafyatis et al., 1991; Paul and Ferl, 1991; Weisshaar et al., 1991; Shen and Ho, 1995; Hiratsuka and Chua, 1997; Sibéril et al., 2001; Basehoar et al., 2004; Chawla and DeMason, 2004; Frangeul et al., 2004; Zhu et al., 2014).

#### The Expression of NtNRAMP3 in Tobacco Is Limited to Specific Tissues

To investigate the tissue-specific expression, tobacco was stably transformed with the *pMDC163::promNtNRAMP3::GUS* construct containing a 1,684 bp promoter region. Out of 30 T1 heterozygous lines with the 3:1 segregation ratio of the transgene, nine homozygous T2 lines were derived and used for experiments (L004, L008, L009, L012, L013, L014, L015, L016, L022).

It was shown that the expression of *NtNRAMP3* was the highest under control conditions (Figure 6). Therefore, the tissue-specific promoter activity was examined in transgenic plants stably expressing the *promNtNRAMP3::GUS* construct in tobacco grown in the control medium.

**Figure 8:**
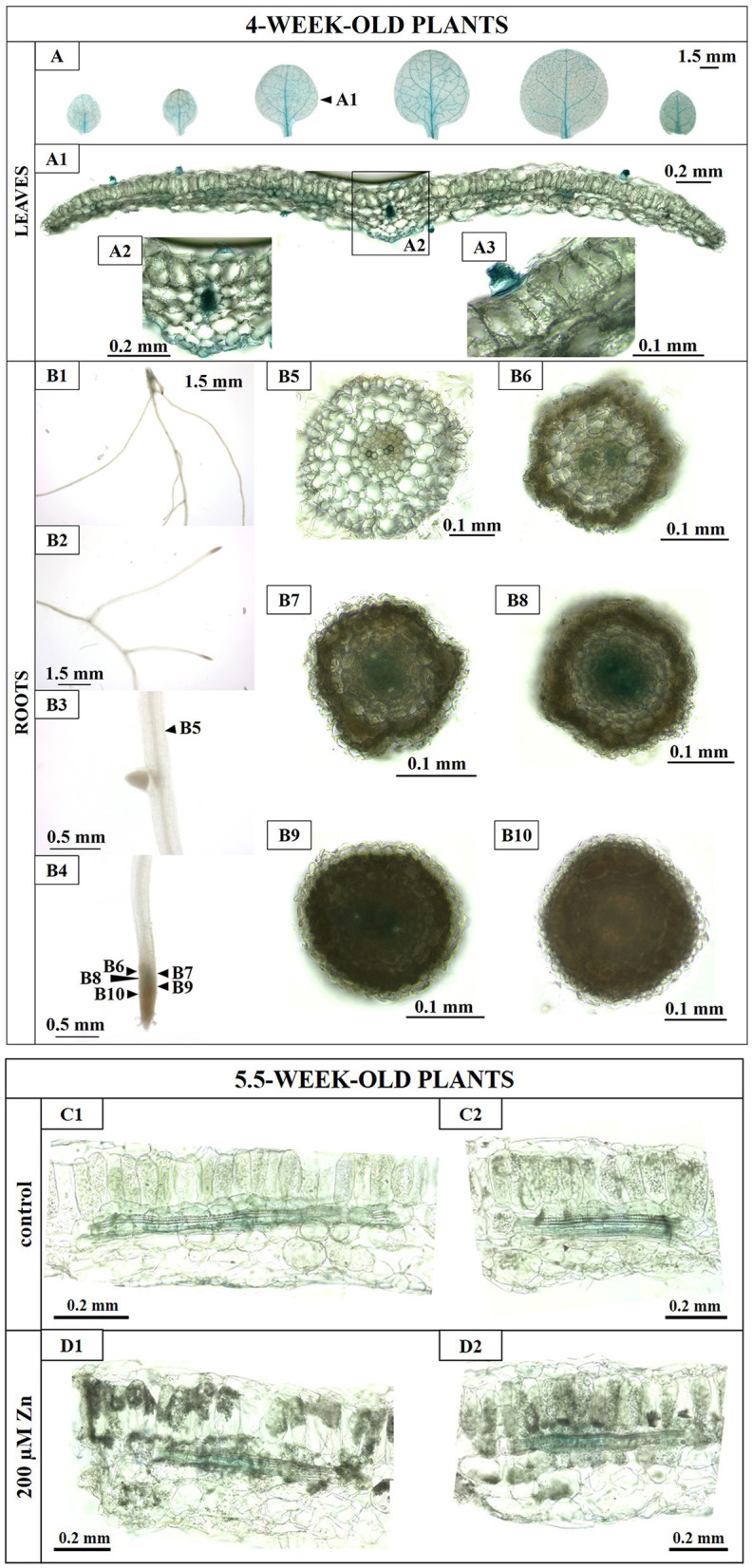
Histochemical GUS assay in transgenic *promNtNRAMP3::GUS* tobacco. **(A–B4)**: *4-week-old plants:* Transgenic *promNtNRAMP3::GUS* tobacco plants were grown for 4 weeks at the control medium ($\frac{1}{4}$ Knop's). **(A)** depicts leaves. **(A1)** Cross-section through the blade of the 3rd leaf (counting from the base). **(A2)** Magnifications of the main vein and surrounding tissues. **(A3)** Fragment of a cross-section of a leaf blade with a trichome. **(B)** Depicts roots. **(B1)** Basal part of the root; **(B2)** middle part of the main root with lateral roots; **(B3)** the middle part of the main root (magnification); **(B4)** apical part of the main root; **(B5)** cross-section through the middle part of the main root; **(B6–B10)** cross-sections through the apical part of the main root. **(C1–D2**): *5.5-week-old plants:* GUS activity in cross-sections through leaf blades of transgenic *promNtNRAMP3::GUS* tobacco plants. Transgenic *promNtNRAMP3::GUS* tobacco plants were grown for 5 weeks in the control medium and then subjected for 3 days to (i) control conditions **(C1,C2)** or 200 μM Zn **(D1,D2)**. Cross-sections through the 2nd leaf (counting from the bottom).

In the leaves of young seedlings, GUS activity was detected primarily in the veins (Figure 8A). However, *NtNRAMP3* expression was also present in the lower epidermis just below the main vein (Figures 8A1,A2), and in the trichomes (Figure 8A3).

In the roots, blue staining was not found (Figures 8B1–B3,B5), except in the apical meristem of the main and lateral roots (Figure 8B4), specifically in the procambium (Figures 8B7–B9), gradually disappearing distally (Figure 8B10). It was also seen in the early stages of differentiation of the vascular bundles (Figure 8B6). In wild-type tobacco, no GUS staining was detected (data not shown).

GUS activity was also examined in cross-sections through 5-week-old leaves from plants exposed for 3 days to 200 μM Zn. No differences were observed between control plants and those exposed to high Zn (Figures 8C1,C2, D1,D2). Blue staining was present only in the vascular bundles.

The promoter activity of the *NtNRAMP3* gene (encoding metal influx transporter) detected mainly in the xylem of leaves indicates that NtNRAMP3 may play an important role in the unloading of minerals carried by vessels from the roots to the leaves, thus supplying the leaf cells with essential micronutrients.

## Discussion

Natural resistance-associated macrophage protein genes are one of the key components of the plant metal homeostasis regulation network. However, they remain largely unknown in such an economically important plant species as tobacco (*Nicotiana tabacum*), also frequently used for phytoremediation of metal-contaminated soil. So far, only *NtNRAMP1* has been cloned from tobacco and preliminary characterization showed that the protein was targeted to the plasma membrane and contributed to Fe uptake (Sano et al., 2012). The present work fills this gap through the functional characterization of the newly cloned *NtNRAMP3* gene. Here, we report that plasma membrane-localized NtNRAMP3 is a broad range metal transporter mediating uptake of Fe, Mn, Co, Cu, Ni, Cd, and also Zn. Its role is likely to primarily maintain cross-homeostasis under control conditions in the leaves.

### *NtNRAMP3* Is a Broad Range Metal Uptake Transporter

The new sequence was assigned to the NRAMP family based on the degree of homology to nucleotide and amino acid sequences characteristic of this family, and also on the structure of the protein. The new sequence was named NtNRAMP3 based on its highest homology to NRAMP3 sequences from other plant species (Figure 1; Table 2). Dendrogram analysis showed the closest relationship between NRAMP3 from all eight plant species used for the study, which formed a separate branch (Figure 1). The highest degree of homology was found between NtNRAMP3 and NRAMP3 proteins from other tobacco species (99.81–98.04%), as well as for NRAMP3 from other plants, such as *S. lycopersicum* or *A. thaliana* (91.14 and 77.18%, respectively) (Table 2).

Homology-based analysis revealed that the overall structure of NtNRAMP3 is conserved, except for the N-terminal and C-terminal regions. These include the presence of 12 TMDs, a glycosylation signal at the C-terminus, and the consensus transport motif (CTM) between VIII and IX TMD (Figure 2; Supplementary File S2), which are indicative of the NRAMP family (Nevo and Nelson, 2006).

Furthermore, the NtNRAMP3 sequence contains essential amino acid residues important for the transport function. These include histidines, which are also believed to contribute to the functional divergence of the NRAMP family (Chaloupka et al., 2005; Ihnatowicz et al., 2014). First, NtNRAMP3 possesses conserved His residues, one within the loop between the II and III TMD and two within VI TMD, including the Met-Pro-His motif (Supplementary File S2). Secondly, in most NRAMP proteins, there are also His residues in a variable arrangement throughout a sequence. Three histidines (H4, H33, and H40) were found in the NtNRAMP3 sequence in the N- terminus, and interestingly, was also present in NRAMP3 from five plant species with the highest homology to NtNRAMP3 (NtomNRAMP3, NaNRAMP3 and NsNRAMP3 proteins, and also in LeNRAMP3 and AtNRAMP3). Two His residues were identified within the loop between VI and VII TMD, in NtNRAMP3 and NtomNRAMP3, NaNRAMP3, and NsNRAMP3 (Supplementary File S2).

The NRAMP proteins carry ions toward the cytoplasm (Nevo and Nelson, 2006). Thus, if a protein is targeted to the plasma membrane it mediates metal uptake, and localization to the tonoplast or the membranes or the Trans-Golgi Network (TGN) indicates involvement in redistribution. Thus, the detected localization of the NtNRAMP3-GFP fusion protein in the plasma membrane (Figure 3) provided indirect evidence for the contribution of the NtNRAMP3 to the uptake of metals. Further pieces of evidence come from yeast experiments. We showed that the NtNRAMP3 protein functions as a Fe, Mn, Co, Cu, Ni, and Cd uptake transporter, as revealed by the functional complementation of the *fet3fet4* and *smf1* yeast mutants, and by the sensitivity tests demonstrating growth inhibition of WT yeast DY1457 expressing the *NtNRAMP3* cDNA (relative to the WT expressing the empty vector), in the presence of elevated concentrations of all tested metals (Figure 4). However, when drawing conclusions, one must remember that sometimes the results of yeast complementation tests are not consistent with the subcellular localization of examined proteins. For example, AtZIP1 is localized to the vacuole of *Arabidopsis* protoplasts, but an expression of *AtZIP1* complemented a yeast mutant defective in plasma membrane Zn uptake. Two possible explanations for this phenomenon have been proposed. First, Zn deficiency-inducible *AtZIP1* might efflux Zn to the cytoplasm of transformed yeast cells which promotes growth on low Zn. Secondly, the plant transporter could be mislocalized to the yeast plasma membrane (Milner et al., 2013). Likewise, AtNRAMP3 and AtNRAMP4 were found to complement the *fet3fet4* Fe uptake mutant although, as being targeted to the tonoplast in plant cells, they participate in metal redistribution (Thomine et al., 2000, 2003; Languar et al., 2005). In our studies, however, the result of the subcellular localization of NtNRAMP3 (plasma membrane, Figure 3) was consistent with the direction of transport resulting from yeast tests (uptake, Figure 4). This supports the conclusion that NtNRAMP3, as a protein with low substrate specificity, is capable of carrying several metals.

For comparison, similar to tobacco, the presence of NRAMP3 in rice was found in the plasma membrane, but AtNRAMP3, TcNRAMP3, and LeNRAMP3 from three other plant species (*A. thaliana, T. caerulescens*, and tomato) were localized in the tonoplast (Bereczky et al., 2003; Thomine et al., 2003; Oomen et al., 2009). The NRAMP proteins with predominant localization in the plasma membrane include NRAMP1 and NRAMP5. This was shown for most of the NRAMP1 proteins known to date (from *A. thaliana, M. truncatula, N. tabacum, O. sativa*, and *Sedum alfredii*) (Thomine et al., 2000; Takahashi et al., 2011; Sano et al., 2012; Tejada-Jiménez et al., 2015; Zhang et al., 2020), except for NRAMP1 from *G. max* or tomato, which was found in intracellular membranes (Bereczky et al., 2003; Qin et al., 2017). Similarly, known NRAMP5 proteins (from *O. sativa, G. max*, and *H. vulgare*) were also targeted in the plasma membrane (Sasaki et al., 2012; Qin et al., 2017).

Although NRAMPs are known as transporters mediating translocation of a broad range of metals (Nevo and Nelson, 2006), the ability of NtNRAMP3 to carry six possible substrates identified in this study (Figure 4) seems to be unique among plants for this class of the proteins. However, the identification of six substrates for NtNRAMP3 resulted from the large scope of yeast-based analyses performed. Until now, usually up to four metals have been tested as possible substrates for an examined protein. For comparison, it has been shown that within the NRAMP3 proteins, the substrate for OsNRAMP3 was Mn, but not Fe or Cd (Yamaji et al., 2013), for AtNRAMP3 or TcNRAMP3 Mn, Fe, Cd, but not Zn (Thomine et al., 2000; Languar et al., 2004; Oomen et al., 2009). Similarly, the number of metals transferred by NRAMP proteins from the other classes (NRAMP1-2, NRAMP4-6) is usually a maximum of 2 to 4 (Thomine et al., 2000; Bereczky et al., 2003; Languar et al., 2004, 2010; Yamaji et al., 2013; Zhang et al., 2020). TjNRAMP4 is an opposite example of an NRAMP protein with high substrate specificity, for which only Ni (but not Fe, Zn, Mn, or Cd) was identified as a substrate (Mizuno et al., 2005).

Considering these data, it seems that NtNRAMP3, capable of carrying up to six different metals, can play a unique role in tobacco. For the proper growth and development of a plant, it is necessary to maintain the correct concentration of many metals (both micro- and macro-elements) (Baxter, 2009; Sperotto et al., 2014; Che et al., 2018). This is due to the interconnected network of regulation of the metal-related genes, for which tissue-specific expression depends on the status of metals in the cells. Underlying mechanisms are poorly understood, especially when we take into account the simultaneous regulation of the level of various metals in a given cell, tissue, or organ. In general, these include the presence of a range of *cis*-acting elements present in the promoter region, as well as the specificity of the transcription factors. Important components of such regulation may also be genes encoding multi-metal transport proteins, such as NtNRAMP3.

### Hypothetical Physiological Role of NtNRAMP3 in Tobacco

#### NtNRAMP3 Might Play a Specific Role in the Regulation of the Balance of Nutrients (Fe, Mn, Co, Cu, and Ni) Under Control Conditions

The uniqueness of the features determining the physiological role of NtNRAMP3 is not only its large number of substrates. The second characteristic important for the protein's function is the expression pattern in leaves and roots. Its highest level was found under control conditions, while reduction took place both at a mineral deficit (Fe, Mn, Co, and Cu) and upon the excess of metals (Fe, Mn, Co, Cu, Ni, and Cd) (Figure 6). Both of these features in association with the *NtNRAMP3* tissue-specific expression found primarily in the vascular bundles of leaves (detected by analysis of prom*NtNRAMP3*-GUS fusion activity; Figure 8) suggest that the encoded metal import protein localized in the plasma membrane (Figure 3) plays a specific role in the regulation of unloading a range of metals (Fe, Mn, Co, Cu, Ni, and Cd identified as NtNRAMP3 substrates) transported through the xylem from the roots to the leaves, primarily under control conditions. Thus, the tissue-specific expression of *NtNRAMP3* in leaves (Figure 8) may contribute to a proper supply of several micronutrients to leaf blades. Downregulation at mineral deficiency and moderate metal excess (Figure 6) support this conclusion. Similarly, OsNRAMP3 is an Mn uptake transporter expressed in the parenchyma cells of the vascular bundles. Experiments with knockout mutants showed that OsNRAMP3 is necessary for the unloading of metal ions from the xylem (Yamaji et al., 2013). Further research is needed to elucidate the molecular mechanisms behind the reduction in *NtNRAMP3* expression under both metal deficit and excess conditions (Figure 6). It cannot be excluded that *NtNRAMP3* is regulated through common or converging regulatory elements in response to such differing metal supply conditions. Similarly, the same direction of changes in expression (increase) in the presence of both excess and deficiency of Fe was demonstrated for *AhNRAMP3* in *Arabidopsis halleri* (Weber et al., 2004). It was hypothesized that *CsMTP6* proteins might participate in the protection of mitochondria from excess Fe and/or in the remobilization from mitochondria under deficiency conditions, however, the molecular background has not been studied.

Bioinformatic analysis showed the presence of various regulatory elements in the *NtNRAMP3* promoter sequence (Supplementary File S5). These include elements responding to metals (MRE1 and IDE2), phytohormones (ABRE and CGTCA-motif regulating responses to abscisic acid and jasmonic acid, respectively), and five light-response elements, pointing to the possible involvement of NtNRAMP3 in a network coordinating responses of tobacco to different factors, which ultimately might regulate several processes. In this study, we only identified the contribution of NtNRAMP3 in the supply of metals to tissues. Future research may indicate a broader function of this protein, for example in linking mineral nutrition with the role of other stimuli in growth and development. For instance, in rice, protein OsNRAMP6 has been assigned a role in regulating the tissue Fe level in connection to tolerance to a fungal pathogen. Such a relationship was given the term “nutritional immunity” (Peris-Peris et al., 2017).

Our results indicate that *NtNRAMP3* plays a role mainly in the leaves. However, Real-Time analysis showed that it was also expressed in the roots, although at a much lower level (Figure 5). Furthermore, the *NtNRAMP3* promoter-derived GUS staining assay revealed that in the roots, *NtNRAMP3* was mainly expressed in the procambium of the apical meristems (Figure 8B4) pointing to its specific contribution to supplying a range of micronutrients to cells that differentiate into the vasculature of the root. In the upper root parts, GUS activity was histochemically undetectable. As a method of lower sensitivity compared with Real-Time, it was probably due to the low expression level of *NtNRAMP3*.

#### Contribution of NtNRAMP3 to Maintain Metal Cross-Homeostasis Specifically at Exposure to 200 μM Zn

The working hypothesis of the conducted research was the assumption that *NtNRAMP3* was involved in the processes determining the cell-specific accumulation of Zn in the mesophyll of tobacco leaves from plants exposed to a high concentration of this metal. It was assumed that *NtNRAMP3* might be an important component of multi-gene regulation of Zn loading into the mesophyll “Zn accumulating cells.” Earlier research led to the identification of two genes important in this process—*NtZIP1-like* and *NtZIP11* (Weremczuk et al., 2020). Both genes encode Zn uptake transporters (Papierniak et al., 2018; Kozak et al., 2019), and their expression occurs specifically in the “Zn accumulating mesophyll cells” of tobacco leaves of plants exposed to high Zn concentrations (Weremczuk et al., 2020). *NtNRAMP3* was the third candidate gene, identified together with *NtZIP1-like* and *NtZIP11* during preliminary studies, which showed increased expression in tobacco leaves 1 to 4 days after exposure to 200 μM Zn (Papierniak et al., 2018).

In this study, however, we showed that at exposure to 200 μM Zn, *NtNRAMP3* unlike *NtZIP1-like* and *NtZIP11*, was not specifically expressed in the groups of “Zn accumulating mesophyll cells” (Figures 8D1,D2). Although, in the presence of 200 μM Zn a substantial increase in the *NtNRAMP3* transcript level was also noted (Figure 7), after administration of moderately enhanced Zn levels (10 or 50 μM) there was little or no increase in expression (Figures 6, 7). Moreover, *NtNRAMP3* expression decreased in response to elevated concentrations of metals, such as Fe, Mn, Co, Cu, and Ni, which are NtNRAMP3 substrates (Figure 6), indicating fine-tuned metal-status-dependent regulation. It is known that exposure of a plant to a high concentration of Zn disrupts the homeostasis of other metals. In response, a range of metabolic pathways is induced to compensate for the generated changes (Sperotto et al., 2014; Ricachenevsky et al., 2015). *NtNRAMP3* may be one of the components of this regulatory response. It seems likely that due to the expression of *NtNRAMP3* in the conducting tissues, an encoded protein that is involved in the influx of metals might contribute to the regulation of the level of Fe, Mn, Co, Cu, and Ni (and also Cd) in leaves from plants exposed to high Zn. It should be noted, however, that the yeast growth assay does not exclude the possibility of transporting Zn *via* NtNRAMP3. As shown in Figure 4M, *NtNRAMP3* did not restore the ability of the high-affinity Zn uptake mutant *zrt1* to grow on a low Zn medium. However, the growth of transformants was impaired on a medium supplemented with high Zn (Figures 4I–L).

Interestingly, a strong increase in *NtNRAMP3* expression was observed only in the 2nd and 3rd pairs of leaves exposed to high Zn (Figure 7), which suggests a specific role of these leaves in Zn accumulation. Transcript levels might have increased due to a direct response to high zinc, or a manifestation of secondary changes to the disturbed balance of other metals within cells.

In conclusion, plants respond to a specific combination of environmental conditions, which should be taken into account in the functional analysis of a metal transporter. It seems that the main function of NtNRAMP3 is to maintain cross homeostasis of Fe, Mn, Co, Cu, Ni (also Cd) under control conditions, by controlling xylem unloading and transfer of metals to neighboring leaf cells. It seems, however, that it may perform the same function when tobacco is exposed to 200 μM Zn, primarily in the 2nd and 3rd pairs of leaves. Further research is necessary to show the underlying mechanisms of metal status-dependent regulation of *NtNRAMP3* expression in tobacco, and also why the response to high Zn is specific to the 2nd and 3rd pairs of leaves only.

## Data Availability Statement

The original contributions presented in the study are included in the article/Supplementary Material, further inquiries can be directed to the corresponding author/s.

## Author Contributions

KK carried out all experiments, performed data analysis, and contributed to writing the manuscript. AP-W contributed to cloning, hydroponic experiments, and expression analysis. MP and AB were involved in the hydroponic experiments and GUS assays. DMA designed the study concept, coordinated the research and supervised experiments, performed data analysis, and wrote the manuscript. All authors read and approved the final version of the manuscript.

## Funding

This work was financially supported by National Science Center, Poland (grant HARMONIA no. NZ3/00527 to DMA).

## Conflict of Interest

The authors declare that the research was conducted in the absence of any commercial or financial relationships that could be construed as a potential conflict of interest.

## Publisher's Note

All claims expressed in this article are solely those of the authors and do not necessarily represent those of their affiliated organizations, or those of the publisher, the editors and the reviewers. Any product that may be evaluated in this article, or claim that may be made by its manufacturer, is not guaranteed or endorsed by the publisher.
