# Supplementary material for: Regulation and Function of Metal Uptake Transporter NtNRAMP3 in Tobacco
Source: Front Plant Sci. 2022 May 31;13:867967. doi: 10.3389/fpls.2022.867967 (PMC9195099; doi:10.3389/fpls.2022.867967)
Supplement: Supplementary file 1 [file Data_Sheet_1.PDF]

Supplementary File S1. Yeast strains used in the study.

NAME	GENOTYPE	SOURCE
**A. Wild-type strains**		
DY1457	MATa; ade1; can1; his3; leu2; trp1; ura3	University of Southampton
BY4742	MATα; his3Δ1; leu2Δ0; lys2Δ0; ura3Δ0	Euroscarf
**B. Mutant strains**		
Δfet3fet4	MATa, trp1 ura3 Δfet3::LEU2 Δfet4::HIS3	University of Southampton
Δsmf1	MATα; ura3Δ0; leu2Δ0; his3Δ1; lys2Δ0; YOL122c::kanMX4	Euroscarf
Δzrt1	MATα; ura3Δ0; leu2Δ0; his3Δ1; lys2Δ0; YGL255w::kanMX4	Euroscarf
