# Supplementary material for: Regulation and Function of Metal Uptake Transporter NtNRAMP3 in Tobacco
Source: Front Plant Sci. 2022 May 31;13:867967. doi: 10.3389/fpls.2022.867967 (PMC9195099; doi:10.3389/fpls.2022.867967)
Supplement: Supplementary file 2 [file Data_Sheet_2.PDF]

Supplementary Figure S2. Alignment of amino acid sequences of selected NRAMP proteins.

Amino acid sequences of 32 NRAMP plant proteins were identified within several databases: ARAMEMNON (*Arabidopsis thaliana*), Solgenomics (*Solanum lycopersicum*), MaizeSequence (*Zea mays*), Phytozome (*Theobroma cacao*), and NCBI (*Nicotiana*). Sequences were aligned with Clustal Omega. The following proteins were provided for the analysis: (i) *Arabidopsis thaliana*: AtNRAMP1, AT1G80830; AtNRAMP2, AT1G47240; AtNRAMP3, AT2G23150; AtNRAMP4, AT5G67330; AtNRAMP5, AT4G18790; AtNRAMP6, AT1G15960; (ii) *Solanum lycopersicum*: LeNRAMP1, Solyc11g018530; LeNRAMP2, Solyc04g078250; LeNRAMP3, Solyc02g092800; LeNRAMP4, Solyc03g116900; (iii) *Zea mays*: ZmNRAMP1, GRMZM2G178190; (iv) *Theobroma cacao*: TheccNRAMP1, Thecc1EG035168; TheccNRAMP2, Thecc1EG034751; TheccNRAMP3, Thecc1EG000729; TheccNRAMP5, Thecc1EG035174; TheccNRAMP6, Thecc1EG027424; (v) *Nicotiana attenuata*: NaNRAMP2, XP_019262545; NaNRAMP3, XP_019228309; NaNRAMP5, XP_019245559; NaNRAMP6, XP_019243869; (vi) *Nicotiana sylvestris*: NsNRAMP2, XP_009760309; NsNRAMP3, XP_009796782; NsNRAMP5, XP_009783885; NsNRAMP6, XP_009774026; (vii) *Nicotiana tabacum*: NtNRAMP2, XP_016477061; NtNRAMP3, NP_001312209 (marked with blue arrow); NtNRAMP5, XP_016434268; NtNRAMP6, XP_016480878; (viii) *Nicotiana tomentosiformis*: NtomNRAMP2, XP_009620156; NtomNRAMP3, XP_009616361; NtomNRAMP5, XP_009620069; NtomNRAMP6, XP_009594426. Transmembrane domains (TMD; marked with grey and numbered from I to XII) were predicted using Phobius software. Histidines are indicated with black background and white letters. Consensus Transport Motif (CTM) is marked with red frame. Dashes indicate gaps.

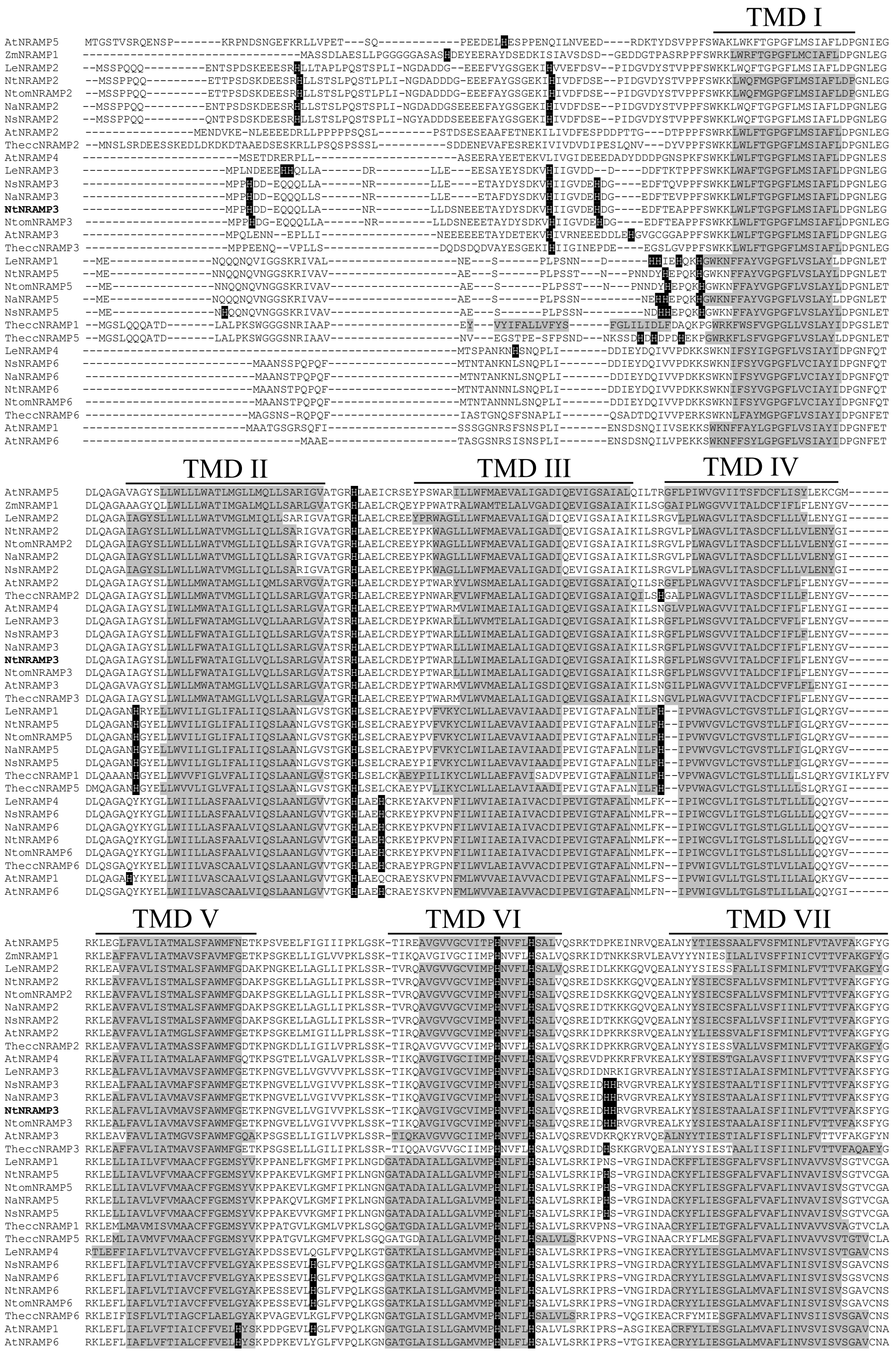
TMD I
TMD II
TMD III
TMD IV
TMD V
TMD VI
TMD VII

TMD VIII — TMD IX

```
AtNRAMP5     -------TKQADSIGLVNAGYYLQEKYGGGVFPILYIWGIGLLAAGQSSTITGTYAGQFIMEGFLDLQMEQWLSAFITRSFAIVPTMFVAIMFNTSEGSL
ZmNRAMP1     -------SKQADSIGLENAGQYLQEKYGTAFFPILYIWAVGLLASGQSSTITGTYAGQFVMGGFLNLRLKKWLRAMITRSFAIIPTMIVALFFDTEDPTM
LeNRAMP2     -------SEQAGSLGLVNAGQFLQDKYGGGLFPILYIWGIGLLAAGQSSTITGTYAGQFIMGGFLDLRLKKWLRALITRSCAIVPTIIVALIFNRSESSL
NtNRAMP2     -------SEQASSLGLVNAGQYLQDKYGGGMFPILYIWGIGLLAAGQSSTITGTYAGQFIMGGFLDLRLKKWLRALITRSCAILPTIVVALIFNRTESSL
NtomNRAMP2   -------SEQASSLGLVNAGQYLQDKYGGGMFPILYIWGIGLLAAGQSSTITGTYAGQFIMGGLLDLRLKKWLRALITRSCAILPTIVVALIFNRTESSL
NaNRAMP2     -------SEQASSLGLVNAGQYLQDKYGGGMFPILYIWGIGLLAAGQSSTITGTYAGQFIMGGFLDLRLKKWLRALITRSCAIVPTIIVALIFNRTESSL
NsNRAMP2     -------SEQASSLGLVNAGQYLQDKYGGGMFPILYIWGIGLLAAGQSSTITGTYAGQFIMGGFLDLRLKKWLRALITRSCAIVPTIIVALIFNRTESSL
AtNRAMP2     -------TEKANNIGLVNAGQYLQEKFGGGLLPILYIWGIGLLAAGQSSTITGTYAGQFIMGGFLNLRLKKWMRAVITRSCAIVPTMIVAIVFNTSEASL
TheccNRAMP2  -------TKQANNIGLVNAGQYLQEKYGGGVFPILYIWGIGLLAAGQSSTITGTYAGQFIMGGFLNLRLKKWLRALITRSFAIVPTIIVALVFNTSEASL
AtNRAMP4     -------TEIADTIGLANAGQYLQDKYGGGFFPILYIWAIGVLAAGQSSTITGTYAGQFIMGGFLNLKMKKWVRALITRSCAIIPTMIVALVFDSSDSML
LeNRAMP3     -------SEIANSIGLENAGQYLQDKYGGGSFPILYIWAIGLLAAGQSSTITGTYAGQFIMGGFLHMRLKKWQRALITRSCAIVPTLIVALAFDTSEKSL
NsNRAMP3     -------SEIANSIGLENAGQYLQEKYGGGVFPILYIWAIGLLAAGQSSTITGTYAGQFIMGGFLHMRLKKWQRALITRSCAIIPTLIVALAFDTSEKSL
NaNRAMP3     -------SEIANSIGLENAGQYLQEKYGGGVFPILYIWAIGLLAAGQSSTITGTYAGQFIMGGFLHMRLKKWQRALITRSCAIIPTLIVALAFDTSEKSL
NtNRAMP3     -------SEIANSIGLENAGQYLQEKYGGGVFPILYIWAIGLLAAGQSSTITGTYAGQFIMGGFLHMRLKKWQRALITRSCAIIPTLIVALAFDTSEKSL
NtomNRAMP3   -------SEIANSIGLENAGQYLQEKYGGGVFPILYIWAIGLLAAGQSSTITGTYAGQFIMGGFLHMRLKKWQRALITRSCAIIPTLIVALAFDTSEKSL
AtNRAMP3     -------TDLANSIGLVNAGQYLQEKYGGGVFPILYIWAIGLLAAGQSSTITGTYAGQFIMGGFLNFKMKKWLRALITRSCAIIPTIIVALVFDSSEATL
TheccNRAMP3  -------TEVADSIGLVNAGQYLQEKYGGGVFPILYIWAIGLLAAGQSSTITGTYAGQFIMGGFLNLRLKKWLRALITRSFAIIPTILVALIFDTSEAAL
LeNRAMP1     DNLSDQNKESCSDITLNSASFLLKNVLGKSS---STVYAIALLASGQSSTITGTYAGQFIMQGFLDLKMKTWLRNLFTRLIAITPSLVVSIIGGSSGAGR
NtNRAMP5     DNLSQENKDSCSDITLNSASFLLKNVLGKSS---STVYAIALLASGQSSTITGTYAGQFIMQGFLDLKMKTWLRNLVTRLIAITPSLVVSIIGGSSGAGR
NtomNRAMP5   DNLSQENKDSCSDITLNSASFLLKNVLGKSS---STVYAIALLASGQSSTITGTYAGQFIMQGFLDLKMKTWLRNLVTRLIAITPSLVVSIIGGSSGAGR
NaNRAMP5     DNLSQQNKDSCSDITLNSASFLLKNVLGKSS---STVYAIALLASGQSSTITGTYAGQFIMQGFLDLKMKTWLRNLITRLIAITPSLVVSIIGGSSGAGR
NsNRAMP5     DNLSQENKDSCSDITLNSASFLLKNVLGKSS---STVYAIALLASGQSSTITGTYAGQFIMQGFLDLKMKTWLRNLITRLIAITPSLVVSIIGGSSGAGR
TheccNRAMP1  NNLSSDDSDRCSNLTLNSASFMLQHVLGKSS---STLYAIALLASGQSSSIADTYAGQFIMQGFLDLKMKKWVRNLVTRSIAIAPSLIVSIIGGSQGAAR
TheccNRAMP5  DNLSSEDQDRCSNLTLDSASFMLQHVLGKSS---STLYAVALLASGQSSTITGTYAGQFIMQGFLDLKMKKWVRNLMTRSIAITPSLIVSIIGGSQGAGR
LeNRAMP4     PSMSPDDQEKCQDLDLNKASFLLKNVLGNWS---SKLFAIALLASGQSSTITGTYAGQYVMQGFLDLRLKPWIRNFLTRCLAIVPSLVVSLIGGSGGAGN
NsNRAMP6     STMTADDREKCEDLDLNKASFLLQNVLGNWS---SKLFAIALLASGQSSTITGTYAGQYVMQGFLDLRLKPWIRNFLTRSLAIVPSLIVSLIGGSAGAGD
NaNRAMP6     STMTADDREKCEDLDLNKASFLLKNVLGNWS---SKLFAIALLASGQSSTITGTYAGQYVMQGFLDLRLKPWIRNFLTRSLAIVPSLIVSLIGGSAGAGD
NtNRAMP6     ATMTPDDREKCEDLDLNKASFLLKNVLGNWS---SKLFAIALLASGQSSTITGTYAGQYVMQGFLDLRLKPWIRNFLTRSLAIVPSLVVSLIGGSAGAGD
NtomNRAMP6   ATMTPDDREKCEDLDLNKASFLLKNVLGNWS---SKLFAIALLASGQSSTITGTYAGQYVMQGFLDLRLKPWIRNFLTRSLAIVPSLIVSLIGGSAGAGD
TheccNRAMP6  SNMNPEDQASCEDLDLNKASFLLRNVLGSWS---SKLFGIALLASGQSSTITGTYAGQYVMQGFLDLRLKPWLRNFLTRCLAIVPSLIVAIIGGSAGAGK
AtNRAMP1     PNLSPEDRANCEDLDLNKASFLLRNVVGKWS---SKLFAIALLASGQSSTITGTYAGQYVMQGFLDLRLEPWLRNLLTRCLAIIPSLIVALIGGSAGAGK
AtNRAMP6     SDLSPEDRASCQDLDLNKASFLLRNVVGKWS---SKLFAIALLASGQSSTITGTYAGQYVMQGFLDLRLEPWLRNFLTRCLAIIPSLIVALIGGSAGAGK
```

TMD X — TMD XI — TMD XII

```
AtNRAMP5     DVLNEWLNILQSMQIPFAVIPLLTMVSNEHIMGVFKIGPSLEKLAWTVA---VFVMMINGYLLL-----------DFFMAEVEGFLVGFLVFGGVVGYISFIIYLVSYRSSQSSSWSS
ZmNRAMP1     DILNEALNVLQSIQIPFALIPLITLVSKEQIMGSFVIGPITKVISWIVT---VFLMLINGYLIL-----------SFYITDVRGALLRSSLCVVLVVYLAFIVYLIVRNTSLYSRLCS
LeNRAMP2     DVLNEWLNVLQSIQIPFALIPLLTLVSKEDIMGTFKLCWSMRHLRQYIQSNPDVVNIVNHYIAERPWPWGSLITEAFFSSEE----VPISLCPNCHNSF------------------
NtNRAMP2     DVLNEWLNVLQSIQIPFALIPLLTLVSKEELMGVFKIGPTLERVAWTVA---ALVMVINGYLLL-----------DFVISEVNGLLFAFLVCAGTAGYIAFILYLISHGGGNVANWFN
NtomNRAMP2   DVLNEWLNVLQSIQIPFALIPLLTLVSKEELMGVFKIGPTLERVAWTVA---ALVMVINGYLLL-----------DFVISEVNGLLFAFLVCAGTAGYIAFILYLISHGGGNVANWFN
NaNRAMP2     DVLNEWLNVLQSIQIPFALIPLLTLVSKKELMGVFKIGPTLERVAWTVA---ALVMVINGYLLL-----------DFVISEVNGLLFAFLVCAGTAGYIAFILYLISHGGGNVANWFN
NsNRAMP2     DVLNEWLNVLQSIQIPFALIPLLTLVSKEELMGVFKIGPTLERVAWTVA---ALVMVINGYLLL-----------DFVISEVNGLLFAFLVCAGTAGYIAFILYLISHGGGNVANWFN
AtNRAMP2     DVLNEWLNVLQSVQIPFALLPLLTLVSKEEIMGDFKIGPILQRIAWTVA---ALVMIINGYLLL-----------DFFVSEVDGFLFGVTVCVWTTAYIAFIVYLISHSNFFPSPWSS
TheccNRAMP2  DILNEWLNVLQSVQIPFALIPLLTLVSKEQVMGVFRIGPILERLAWTVA---ALVIIINGYLLL-----------DFFVSEVKGPLFALFICSWTAAYIAFIVYLVARGGGLPSTWFS
AtNRAMP4     DELNEWLNVLQSVQIPFAVIPLLCLVSNEQIMGSFKIQPLVQTISWIVA---ALVIAINGYLMV-----------DFFSGAATNLILLVPVIIFAIAYVVFVLYLISRGLTYTP-WQL
LeNRAMP3     DVLNEWLNVLQCVQIPFALIPLLCLVSREEIMGVFKIGSTMKVISWLVA---VLVMLINGYLLM-----------DSLSSAVSGMLFTSVVVAFTGAYIAFIVYLISRGITFPN-WFV
NsNRAMP3     DVLNEWLNVLQSVQIPFALIPLLCLVSKEEIMGVFKIGSTLKVISWLVA---ALVIMINGYLLM-----------DSLSSAVSGVLFTSVVFAFTGGYVAFIVYLILRGITFPD-WFV
NaNRAMP3     DVLNEWLNVLQSVQIPFALIPLLCLVSKEEIMGVFKIGSTLKVISWLVA---ALVILINGYLLM-----------DSLSSAVSGVLFTSVVFAFTGGYVAFIVYLILRGITFPN-WFV
NtNRAMP3     DVLNEWLNVLQSVQIPFALIPLLCLVSKEEIMGVFKIGSTLKVISWLVA---ALVILINGYLLM-----------DSLSSAVSGVLFTSVVFAFTGGYVAFIVYLILRGITFPN-WFV
NtomNRAMP3   DVLNEWLNVLQSVQIPFALIPLLCLVSKEEIMGVFKIGSTLKVISWLVA---ALVILINGYLLM-----------DSLSSAVSGVLFTSVVFAFTGGYVAFIVYLILRGITFPN-WFV
AtNRAMP3     DVLNEWLNVLQSIQIPFALIPLLCLVSKEQIMGSFKIGPLYKTIAWLVA---ALVIMINGYLLL-----------EFFSNEVSGIVYTGFVTLFTASYGAFILYLIARGITFTP-WPF
TheccNRAMP3  DVLNEWLNVLQSIQIPFALIPLLCLVSKEQIMGTFKIGPVLKTVAWLVA---VLVIVINGYLLV-----------DFFSSEVTGVMFASVVFAFTGAYLAFIVYLVSRGFTLPA-WRC
LeNRAMP1     --LIIIASMILSFELPFALIPLLKFSSSSTKLGPHKNSIYIIVISWILG---LGIIGINIYYLSTAFVG-WLI--SNNLPKVGNVLIGIVVFPLMAIYILAVIYLMFRKDKVVTYIDP
NtNRAMP5     --LIIIASMILSFELPFALIPLLKFSSSSTKLGPHKNSIYIIVISWILG---LGIIGINIYYLTTAFVD-WLI--HNNLPKVGNVFIGIVVFPLMAIYILSVIYLMFRKDTVVTYIDP
NtomNRAMP5   --LIIIASMILSFELPFALIPLLKFSSSSTKLGPHKNSIYIIVISWILG---LGIIGINIYYLTTAFVD-WLI--HNNLPKVGNVFIGIVVFPLMAIYILSVIYLMFRKDTVVTYIDP
NaNRAMP5     --LIIIASMILSFELPFALIPLLKFSSSSTKLGPHKNSIYIIVISWILG---LGIIGINIYYLTTAFVD-WLI--HNNLPKVGNVFIGIVVFPLMAIYVLAVIYLMFRKDTVVTYIDP
NsNRAMP5     --LIIIASMILSFELPFALIPLLKFSSSSTKLGPHKNSIYIIVISWILG---LGIIGINIYYLTTAFVD-WLI--HNNLPKVGNVFIGIVVFPLMAIYILAVIYLMFRKDTVVTYIDP
TheccNRAMP1  --LIIISSMILSYVLPFTFIPLLKFSNSSAKMGPHKNSIYIIVISWILG---LGMFGINVYYLITTFVG-WLI--HDDLPKVGNVFIGIIVFPLMAIYILSMIYLTFRKDTVVTYIEP
TheccNRAMP5  --LIIIASMILSFELPFALIPLLKFSSSSTKMGPYKNSIYIIVISWILG---LGIIGINVYYLSTAFVG-WLI--HNDLPKVGNVFIGIIVFPLMAIYILSVIYLTFRKDTVVTYIEP
LeNRAMP4     --LIIIASMILSFELPFALIPLLKFTSSKTKMGSHANPVAVSAATWVIG---TLIMGINIYYLAEKLIT-SIK--DGHLGKASKVLCGLLGASCLLGYLSSILYLAFRRNKEGTHLLA
NsNRAMP6     --LIIIASMILSFELPFALIPLLKFTSSKTKMGSHVNPVAVSAATWVIG---TLIMGINIYYLAEKLVT-SLK--DSHLGKAVKVLCGILGTLCLLVYLCSILYLAIRKNKESTHLLA
NaNRAMP6     --LIIIASMILSFELPFALIPLLKFTSSKTKMGSHVNPVAVSAATWVIG---TLIMGINIYYLAEKLVS-SLK--DSHLGKAVKVLCGILGALCLLVYLCSILYLAIRKNKESTHLLA
NtNRAMP6     --LIIIASMILSFELPFALIPLLKFTSSKTKMGSHVNPIAVSGATWLIG---TLIMGINIYYLAEKLVT-SLK--DSHLGKAVKVLCGILGALCLLVYLCSILYLAIRKNKESTHLLA
NtomNRAMP6   --LIIIASMILSFELPFALIPLLKFTSSKTKMGSHVNPIAVSGATWLIG---TLIMGINIYYLAEKLVT-SLK--DSHLGKAVKVLCGILGALCLLVYLCSILYLAIRKNKESTHLLA
TheccNRAMP6  --LIIIASMILSFELPFALIPLLKFTSSETKMGEHANSTAVSAIAWIIG---SLIMGINIYYLVTSFIH-FLL--QSHWKLIAVVFLGIFGFVGVAIYLAAVAYLVFRPNKKATHLLA
AtNRAMP1     --LIIIASMILSFELPFALVPLLKFTSCKTKMGSHVNPMAITALTWVIG---GLIMGINIYYLVSSFIK-LLI--HSHMKLILVVFCGILGFAGIALYLAAIAYLVFRKNRVATS---
AtNRAMP6     --LIIIASMILSFELPFALVPLLKFTSSKTKMGSHANSLVISSVTWIIG---GLIMGINIYYLVSSFIK-LLL--HSHMNLVAIVFLGVLGFSGIATYLAAISYLVLRKNRESSS---
```

```
AtNRAMP5     LEMSERVVSTET-------------------------------------
ZmNRAMP1     ST-SKSS------------------------------------------
LeNRAMP2     -------------------------------------------------
NtNRAMP2     LLRTKGYSYAGQ-------------------------------------
NtomNRAMP2   LLRTKGYSYAGQ-------------------------------------
NaNRAMP2     LLRTKGYSYAGQ-------------------------------------
NsNRAMP2     LLRTKGYSYAGQ-------------------------------------
AtNRAMP2     SSIELPKRVSVSNS-----------------------------------
TheccNRAMP2  I--ELSKRFSATGS-----------------------------------
AtNRAMP4     VASSHKEPQRD-DE-----------------------------------
LeNRAMP3     KNKSISSIDN---------------------------------------
NsNRAMP3     KNKSITSIEN---------------------------------------
NaNRAMP3     KNKSISSIEN---------------------------------------
NtNRAMP3     KNKSITSIEN---------------------------------------
NtomNRAMP3   KNKSITSIEN---------------------------------------
AtNRAMP3     KAESSH-------------------------------------------
TheccNRAMP3  LVQSKQTQGIE--------------------------------------
LeNRAMP1     IKDD-----HMENGINSME---LVDRVPYREDLADIPLPQ---------
NtNRAMP5     IKDNPNAQRYMEDGINNME---LVDRVPYREDLADIPLPQ---------
NtomNRAMP5   IKDNPNAQRYMEDGINNME---LVDRVPYGEDLADIPLPQ---------
NaNRAMP5     IKDNPNAQRYMEDGTNNVE---LVDRVPYREDLADIPLPQ---------
NsNRAMP5     IKDNPNAQRYMEDGTNNVE---LVDRVPYREDLADIPLPQ---------
TheccNRAMP1  EMNDPAAQARMESGLSNPDGSFRADDVPYGQDLADIPLPE*--------
TheccNRAMP5  DKNDPTAQARMESGLSNLDGSFRADNVPYRQDLADIPLPE*--------
LeNRAMP4     LTGQEGLQVS------------E-LNNLPREDILRMQLPQQRTTN----
NsNRAMP6     LTGQKVYKFL------------N-QTIYLERTFXRMQLPQQRTTN----
NaNRAMP6     LTGQEGLQVS------------E-SNNLPREDILRMQLPQQRTTN----
NtNRAMP6     LTGQEGLQVS------------E-SNNLPREDILRMQLPQQRTTN----
NtomNRAMP6   LTGQEGLQVS------------E-SNNLPREDILRMQLPQQRTTN----
TheccNRAMP6  LTTPDSRHMVNGSG------RTS-VDCLPREDIVSMQLPQRREQTEDMD
AtNRAMP1     --------LLISRD------SQN-VETLPRQDIVNMQLPCRVSTSDVD-
AtNRAMP6     ------THFLDFSN------SQT-EETLPREDIANMQLPNRVAVIGDLN
```
