# Supplementary material for: Regulation and Function of Metal Uptake Transporter NtNRAMP3 in Tobacco
Source: Front Plant Sci. 2022 May 31;13:867967. doi: 10.3389/fpls.2022.867967 (PMC9195099; doi:10.3389/fpls.2022.867967)
Supplement: Supplementary file 3 [file Data_Sheet_3.PDF]

Supplementary File S3. Appearance of 5.5-week-old tobacco plants used for *NtNRAMP3* expression analysis.

5-week-old (3 weeks on agar plates and 2 weeks in hydroponics) wild-type (WT) tobacco plants were exposed to different metal regimes: (i) control conditions (¼ Knop's medium; (A); (ii) metal excess: 200 µM Fe (C) or 100 µM Mn (E) or 20 µM Co (H) or 20 µM Cu (K-L) or 30 µM Ni (F) or 4 µM Cd (I); (iii) metal deficiency: Fe (B) or Mn (D) or Co (G) or Cu (J) for 3 days. Plants exposed to Zn deficiency and to 50 µM Zn did not differ from the control ones. Images C1, F1, H1 and K1 depicts magnifications of leaves which differ from leaves of control plants (A1). Images L1 and L2 shows magnifications of apical root parts which differ from apical root parts of control plants.

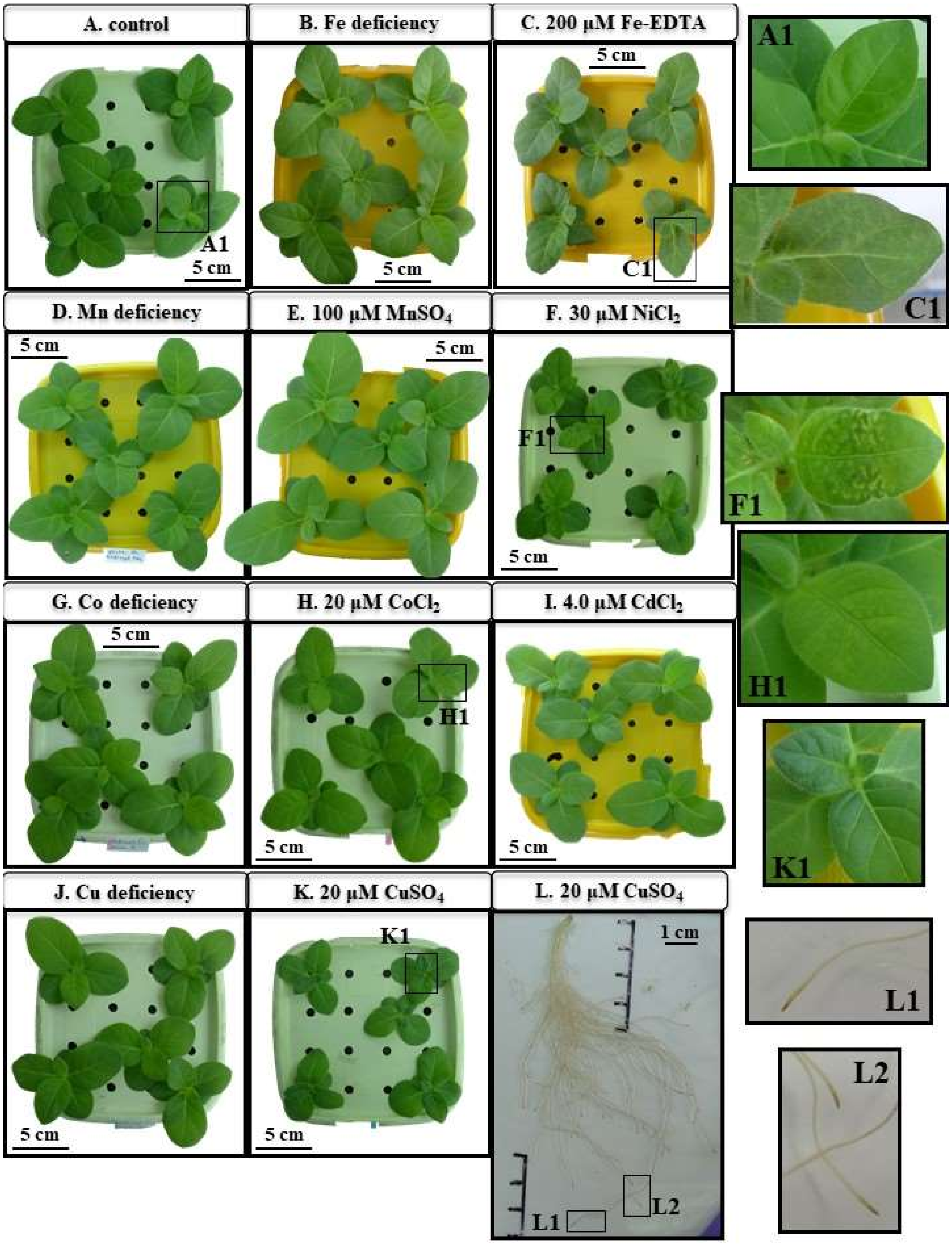

Table 1. Comparison of appearance of tobacco plants grown on different metal regimes. In comparison with control plants (plants grown on ¼ Knop's medium): + - larger plants; ++ - the largest plants; - - smaller plants; -- - the smallest plants; = - the same size of the plants (as control plants).

MEDIUM	SIZE OF THE PLANTS	DIFFERENCES IN APPEARANCE
**control (¼ Knopa)**	plants of standard size and appearance	
**Fe deficiency**	+	no changes in appearance on leaves and roots
**200 µM Fe-EDTA**	=	discolorations of a leaf tissue located between veins
**Mn deficiency**	+	no changes in appearance on leaves and roots
**100 µM $MnSO_4$**	++	no changes in appearance on leaves and roots
**30 µM $NiCl_2$**	-	large chlorosis on youngest leaves
**Co deficiency**	=	no changes in appearance on leaves and roots
**20 µM $CoCl_2$**	=	chlorosis on youngest leaves
**4 µM $CdCl_2$**	=	no changes in appearance on leaves and roots
**Cu deficiency**	=	no changes in appearance on leaves and roots
**20 µM $CuSO_4$**	--	darker green colour of the leaves and embossings of a leaf tissue located between larger veins of the youngest leaves; brown colour of the apical parts of the roots
