# Supplementary material for: Regulation and Function of Metal Uptake Transporter NtNRAMP3 in Tobacco
Source: Front Plant Sci. 2022 May 31;13:867967. doi: 10.3389/fpls.2022.867967 (PMC9195099; doi:10.3389/fpls.2022.867967)
Supplement: Supplementary file 4 [file Data_Sheet_4.PDF]

Supplementary File S4. Stability of *NtPP2A*.

SAMPLE NAME		MEAN Cp
**A. Age-dependent *NtNRAMP3* expression**		
**4 weeks**	**R**	22,424
	**L**	22,795
**6 weeks**	**R**	21,140
	**L**	22,540
**9 weeks**	**AR**	22,318
	**BR**	22,681
	**S**	21,720
	**YL**	22,439
	**OL**	22,413

SAMPLE NAME		MEAN Cp	SAMPLE NAME		MEAN Cp
**B. Metal-induced *NtNRAMP3* expression**					
	**AR**	27,484		**AR**	20,516
**200 µM Fe**	**BR**	26,559	**control**	**BR**	20,860
	**L**	26,184		**L**	22,353
	**AR**	27,370		**AR**	21,367
**Fe deficiency**	**BR**	27,723	**control**	**BR**	22,268
	**L**	24,932		**L**	23,349
	**AR**	25,897		**AR**	20,516
**100 µM Mn**	**BR**	24,736	**control**	**BR**	20,963
	**L**	22,760		**L**	22,353
	**AR**	23,144		**AR**	20,813
**Mn deficiency**	**BR**	24,736	**control**	**BR**	21,213
	**L**	22,760		**L**	22,397
	**AR**	26,430		**AR**	21,926
**20 µM Co**	**BR**	26,038	**control**	**BR**	21,538
	**L**	27,893		**L**	23,896
	**AR**	25,488		**AR**	22,112
**Co deficiency**	**BR**	25,582	**control**	**BR**	21,727
	**L**	25,142		**L**	23,896
	**AR**	31,541		**AR**	21,926
**20 µM Cu**	**BR**	23,777	**control**	**BR**	21,538
	**L**	24,542		**L**	23,019
	**AR**	28,386		**AR**	22,083
**Cu deficiency**	**BR**	26,941	**control**	**BR**	22,011
	**L**	24,202		**L**	23,235
	**AR**	26,219		**AR**	22,453
**30 µM Ni**	**BR**	25,625	**control**	**BR**	21,384
	**L**	25,382		**L**	23,395
	**AR**	22,720		**AR**	21,934
**4 µM Cd**	**BR**	22,657	**control**	**BR**	22,196
	**L**	23,911		**L**	23,601
	**AR**	20,585		**AR**	19,781
**Zn deficiency**	**BR**	20,692	**control**	**BR**	20,765
	**L**	22,005		**L**	22,826
	**AR**	20,152		**AR**	20,532
**replete**	**BR**	20,275	**control**	**BR**	21,300
	**L**	22,081		**L**	22,476
	**AR**	20,026		**AR**	20,568
**50 µM Zn**	**BR**	20,664	**control**	**BR**	20,651
	**L**	21,998		**L**	21,692
	**L1**	21,095		**L1**	20,787
	**L2**	21,035		**L2**	19,943
**control**	**L3**	21,341	**50 µM Zn**	**L3**	21,028
	**L4**	22,114		**L4**	21,286
	**L5**	21,254		**L5**	21,545
	**L1**	20,260		**L1**	23,477
	**L2**	20,723	**200 µM Zn**	**L2**	22,034
**10 µM Zn**	**L3**	20,835		**L3**	25,933
	**L4**	21,347		**L4**	21,544
	**L5**	21,352			
