# Supplementary material for: Regulation and Function of Metal Uptake Transporter NtNRAMP3 in Tobacco
Source: Front Plant Sci. 2022 May 31;13:867967. doi: 10.3389/fpls.2022.867967 (PMC9195099; doi:10.3389/fpls.2022.867967)
Supplement: Supplementary file 5 [file Data_Sheet_5.pdf]

Supplementary File S5. *NtNRAMP3* promoter analysis.

A. *NtNRAMP3* promoter sequence

Sequence of *NtNRAMP3* promoter (*promNtNRAMP3* + 5’UTR + START codon) located in the scaffold AWOK01S026429 (position 12116…13800):

AGAATCTGCGAGCATCTCAAAGGAATCTATGGAATGCTCGGGAAGAAGCGAATACCACGTCAAGGCTCCCCTCGT
GAGTGTCTCCTCGAATTTCTTCAGCAAAACAGATTCAATCTCGTGGGGAGCTAAAATCGTTCCCCTTCACCACCG
TTGCATAGGTGATAATATGTTTATGAGAGTCCGAAGTCCCTCAAGATTGTGTGTATTGTTAACACCATTATTTGC
CATTTTCTGTAATTTTTGCTAAGAACATATAATCAAAACATGTTAGTAAAAAAGACAAGGATCAACTTAATTACA
CGATTTTCTAGGCCCCACGATGGACGCCAAACTATTTGCCCGTAAAACGATACAGTTGAATTTATACGTACTTTC
TAGACAAGTGAATTAATTCGATCCTGAAATAATAAAAGAATTGAATAAATATGCAATATTTAGCCTTAATATGAA
AATGAAATAGCAGAAACCGTAGTTTCGGGAGCAGGGCTTCCGGGCACAACATCGATGAAATCAAAAGGCGAGAGG
ATAAAGTTGTATAAAGCTTTTAATATATTGTAGCGTAAGTATGCTAGAAAATGCGTCCCTTTTACAATGATAACA
GAGCTCATTATTTATAGTTGCATCTAGGGAATAAGGTCCTAGGATTATGTCCTTCTTTAATATGAGGGCTATTAA
AGAATGTGTAACGGCAGGCATGAATGATAAATTTTTTGTAACGGCCAATGTACTAAATACTGTAGAATATTCTTC
ATTAAATGCTATAGTGACAGGCATTTATTGCATATTTATGATCATTATTCTTTTTCGGTAATAAATGAAACAGTT
GTCTTCGGATTTAGCTATCCTCTGCCTTCGGCTTCACGTGTAACTCTCTTATACAATTACTTAACATACCATATT
TTACCCTATACATATAGATTCATAAAAAATATAAAACACAGAAAAACTTTACTGCATTTGCACCAAAAAGGTAAT
GGGAAGGATCAAAGTATTTAATTTTTTGTGTTAATTTGGAATATCCGTTATTTATTTTTCAAAGAAAAAATTCAA
ATATTCAAAATTACAGTAAAATAAATATAAAACCTTCAAAATAATTATCGTCCAAGAGATAGGAAAAAGTGAAAA
ACAATTTAGTCTTAGTTTCACAATGTCAGACTCTCCCTGTTCTTCCCCTCATCACTAAATAAGAAAAAGTCAATC
GATCTCTTATGCTTCTATCTCAAATTAAGAAAAATTTCAATTATATCTATTTTAAGCGGACTTATTTCATTCAAA
TAACCAGAAAAAAGATTTTCCAAAACTAGGGGTTGTATCCTTTACCGCTTACTAAACTAAGAAGACATTGATAAA
TCCAAGATTATAAATATAACATCGATAAATAAACATAATTCCAATTATAGTTGCTATACTTTTGGCTTTACATTG
TATTTTATTTTTTAGCTAAATTCATACTCATTTCAAAGTGGAAAAAAGGAAAAACAAAATAAAAGGAGAAAGGAA
ATTGGGGTGCAAATAGAAAGCGGTAGCGTAATGGTAGTTTTCAAAAATCATTCGTCTTATCGTCTGAAAAGAAAA
ACCAGAAAAAGATCCAAAGCCTCCACTATAAAACAACATCACTTCGCACAAACCGAACTCACCTGTACCTTTTAA
C**CGCTAATATCCTCTTTACAGATTTCTCTTCTAA**ATG

AGAATC – primers used for *promNtNRAMP3* amplification (sequence of the forward primer and complementary sequence of the reverse primer)
ATG – START codon (not amplified with *NtNRAMP3* promoter sequence)

Sequence length: 1684 bp

B. *In situ* analysis of the *NtNRAMP3* promoter

In situ analysis of the *NtNRAMP3* promoter sequence was performed by PlantCARE software (http://bioinformatics.psb.ugent.be/webtools/plantcare/html/) to identify the *cis*-acting elements within its region. For the analysis, a 1684 bp sequence of the *NtNRAMP3* promoter (counting from the START codon upstream) was used.

Table 1. Cis-acting regulatory elements present in *NtNRAMP3* promoter sequence.

***cis*-acting regulatory element**	**Sequence**	**Position in *NtNRAMP3* promoter sequence**	**Function**	**Reference**
**METAL RESPONSIVE SEQUENCES**				
**MRE1**	TGC(A/G)C(T/G/C/A)C	959	sequence of the Metal Response Element 1	1
**IDE2**	CA(A/C)G(C/T)(A/C)A	56	sequence of the Iron Deficiency Response Element 2	2
**GENERAL REGULATORY ELEMENTS**				
TATA-box	ATTATA	1394	core promoter element around -30 of transcription start site	3
**CAAT-box**	(C)C(A)AAT	multi-position	common *cis*-acting element in promoter and enhancer regions from *Pisum sativum* or *Arabidopsis thaliana*	4
**PHYTOHORMONE RESPONSIVE SEQUENCES**				
ABRE	ACGTG	861	*cis*-acting element involved in the abscisic acid responsiveness from *Arabidopsis thaliana*	5
CGTCA-motif	CGTCA	58	*cis*-acting regulatory element involved in the MeJA-responsiveness from *Hordeum vulgare*	6
**LIGHT RESPONSIVE SEQUENCES**				
BOX4	ATTAAT	387	part of a conserved DNA module involved in light responsiveness from *Petroselinum crispum*	7, 8
G-box	CACGTC	56	*cis*-acting regulatory element involved in light responsiveness from *Zea mays*	9
GATA-motif	GATAGGG	1108	part of a light responsive element from *Arabidopsis thaliana*	10
TCCC-motif	TCTCCCT	1157	part of a light responsive element from *Spinacia oleracea*	11
chs-CMA1a	TTACTTAA	882	part of a light responsive element from *Daucus carota*	12
**STRESS RESPONSIVE SEQUENCES**				
ARE	AAACCA	1574	*cis*-acting regulatory element essential for the anaerobic induction from *Zea mays*	13

promNtNRAMP3

IDE2

\+ AGAATCTGCG AGCATCTCAA AGGAATCTAT GGAATGCTCG GGAAGAAGCG AATACCACGT CAAGGCTCCC 70

\+ CTCGTGAGTG TCTCCTCGAA TTTCTTCAGC AAAACAGATT CAATCTCGTG GGGAGCTAAA ATCGTTCCCC 140

\+ TTCACCACCG TTGCATAGGT GATAATATGT TTATGAGAGT CCGAAGTCCC TCAAGATTGT GTGTATTGTT 210

\+ AACACCATTA TTTGCCATTT TCTGTAATTT TTGCTAAGAA CATATAATCA AAACATGTTA GTAAAAAAGA 280

\+ CAAGGATCAA CTTAATTACA CGATTTTCTA GGCCCCACGA TGGACGCCAA ACTATTTGCC CGTAAAACGA 350

\+ TACAGTTGAA TTTATACGTA CTTTCTAGAC AAGTGAATTA ATTCGATCCT GAAATAATAA AAGAATTGAA 420

\+ TAAATATGCA ATATTTAGCC TTAATATGAA AATGAAATAG CAGAAACCGT AGTTTCGGGA GCAGGGCTTC 490

\+ CGGGCACAAC ATCGATGAAA TCAAAAGGCG AGAGGATAAA GTTGTATAAA GCTTTTAATA TATTGTAGCG 560

\+ TAAGTATGCT AGAAAATGCG TCCCTTTTAC AATGATAACA GAGCTCATTA TTTATAGTTG CATCTAGGGA 630

\+ ATAAGGTCCT AGGATTATGT CCTTCTTTAA TATGAGGGCT ATTAAAGAAT GTGTAACGGC AGGCATGAAT 700

\+ GATAAATTTT TTGTAACGG**C CAAT**GTACTA AATACTGTAG AATATTCTTC ATTAAATGCT ATAGTGACAG 770

\+ GCATTTATTG CATATTTATG ATCATTATTC TTTTTCGGTA ATAAATGAAA CAGTTGTCTT CGGATTTAGC 840

\+ TATCCTCTGC CTTCGGCTTC ACGTGTAACT CTCTTATACA ATTACTTAAC ATACCATATT TTACCCTATA 910

MRE1

\+ CATATAGATT CATAAAAAAT ATAAAACACA GAAAAACTTT ACTGCATTTG CACCAAAAAG GTAATGGGAA 980

\+ GGATCAAAGT ATTTAATTTT TTGTGTTAAT TTGGAATATC CGTTATTTAT TTTTCAAAGA AAAAATT**CAA** 1050

\+ **AT**ATTCAAAA TTACAGTAAA ATAAATATAA AACCTTCAAA ATAATTATCG TCCAAGAGAT AGGAAAAAGT 1120

\+ GAAAAACAAT TTAGTCTTAG TTTCACAATG TCAGACTCTC CCTGTTCTTC CCCTCATCAC TAAATAAGAA 1190

\+ AAAGTCAATC GATCTCTTAT GCTTCTATCT **CAAAT**TAAGA AAAATTTCAA TTATATCTAT TTTAAGCGGA 1260

\+ CTTATTTCAT T**CAAAT**AACC AGAAAAAAGA TTTTCCAAAA CTAGGGGTTG TATCCTTTAC CGCTTACTAA 1330

\+ ACTAAGAAGA CATTGATAAA TCCAAGATTA TAAATATAAC ATCGATAAAT AAACATAATT **CCAAT**TATAG 1400

\+ TTGCTATACT TTTGGCTTTA CATTGTATTT TATTTTTTAG CTAAATTCAT ACTCATTTCA AAGTGGAAAA 1470

\+ AAGGAAAAAC AAAATAAAAG GAGAAAGGAA ATTGGGGTG**C AAAT**AGAAAG CGGTAGCGTA ATGGTAGTTT 1540

+ TCAAAAATCA TTCGTCTTAT CGTCTGAAAA GAAAAACCAG AAAAAGATCC AAAGCCTCCA CTATAAAACA 1610

+ ACATCACTTC GCACAAACCG AACTCACCTG TACCTTTTAA CCGCTAATAT CCTCTTTACA GATTTCTCTT 1680

+ CTA

References:

1. Li, Y., et al., *Spatial-temporal analysis of zinc homeostasis reveals the response mechanisms to acute zinc deficiency in Sorghum bicolor*. New Phytologist, 2013. 200(4): p. 1102-1115.
2. Ogo, Y., Kobavashi, T., Itai, R.N., Nakanishi, H., Kakei, Y., Takahashi, M., Toki, S., Mori, S., Nishizawa, N.K. 2008. *A Novel NAC Transcription Factor, IDEF2, That Recognizes the Iron Deficiency-responsive Element 2 Regulates the Genes Involved in Iron Homeostasis in Plants*. J. Biol. Chem. 283, 13407-13417
3. Basehoar, A.D., Zanton, S., Pugh, B. 2004. *Identification and Distinct Regulation of Yeast TATA Box-Containing Genes*. Cell, 116(5):699-709.
4. Frangeul, L., Glaser, P., Rusniok, C., Buchrieser, C., Duchaud, E., Dehoux, P., Kunst, F. 2004. *CAAT-Box, contigs-Assembly and Annotation Tool-Box for genome sequencing projects*. Bioinformatics, 20(5):790–797
5. Shen, Q., Ho, T.H. 1995. *Functional dissection of an abscisic acid (ABA)-inducible gene reveals two independent ABA-responsive complexes each containing a G-box and a novel cis-acting element*. The Plant Cell. 7, 295-307.
6. Fink, J.S., et al., *The CGTCA sequence motif is essential for biological activity of the vasoactive intestinal peptide gene cAMP-regulated enhancer*. Proceedings of the National Academy of Sciences, 1988. 85(18): p. 6662-6666.
7. Hiratsuka, K., Chua, N.H. 1997. *Light regulated transcription in higher plants*. J. Plant. Res. 110(1), 131–139.
8. Weisshaar, B., Block, A., Armstrong, G.A., Herrmann, A., Schulze-Lefert, P., Hahlbrock, K. 1991. *Regulatory elements required for light-mediated expression of the Petroselinum crispum chalcone synthase gene*. Symp. Soc. Exp. Biol. 45,191-210
9. Sibéril, Y., P. Doireau, and P. Gantet, *Plant bZIP G-box binding factors. Modular structure and activation mechanisms.* European Journal of Biochemistry, 2001. 268(22): p. 5655-5666.
10. Chawla, R. and D.A. DeMason, *Molecular expression of PsPIN1, a putative auxin efflux carrier gene from pea (Pisum sativum L.).* Plant growth regulation, 2004. 44(1): p. 1-14.
11. Lafyatis, R., et al., *Sequence specific protein binding to and activation of the TGF-β3 promoter through a repeated TCCC motif.* Nucleic acids research, 1991. 19(23): p. 6419-6425.
12. Zhu, M., et al., *Characterization of the promoter of Artemisia annua amorpha-4, 11-diene synthase (ADS) gene using homologous and heterologous expression as well as deletion analysis.* Plant molecular biology reporter, 2014. 32(2): p. 406-418.
13. Paul, A.-L. and R.J. Ferl, *In vivo footprinting reveals unique cis-elements and different modes of hypoxic induction in maize Adh1 and Adh2.* The Plant Cell, 1991. 3(2): p. 159-168.
